# Draft Genome Sequencing of *Giardia intestinalis* Assemblage B Isolate GS: Is Human Giardiasis Caused by Two Different Species?

**DOI:** 10.1371/journal.ppat.1000560

**Published:** 2009-08-21

**Authors:** Oscar Franzén, Jon Jerlström-Hultqvist, Elsie Castro, Ellen Sherwood, Johan Ankarklev, David S. Reiner, Daniel Palm, Jan O. Andersson, Björn Andersson, Staffan G. Svärd

**Affiliations:** 1 Department of Cell and Molecular Biology, Karolinska Institutet, Stockholm, Sweden; 2 Department of Cell and Molecular Biology, BMC, Uppsala University, Uppsala, Sweden; 3 Centre for Microbiological Preparedness, Swedish Institute for Infectious Disease Control, Solna, Sweden; 4 The Burnham Institute for Medical Research, La Jolla, California, United States of America; 5 Department of Evolution, Genomics and Systematics, EBC, Uppsala University, Uppsala, Sweden; University of Virginia Health System, United States of America

## Abstract

*Giardia intestinalis* is a major cause of diarrheal disease worldwide and two major *Giardia* genotypes, assemblages A and B, infect humans. The genome of assemblage A parasite WB was recently sequenced, and the structurally compact 11.7 Mbp genome contains simplified basic cellular machineries and metabolism. We here performed 454 sequencing to 16× coverage of the assemblage B isolate GS, the only *Giardia* isolate successfully used to experimentally infect animals and humans. The two genomes show 77% nucleotide and 78% amino-acid identity in protein coding regions. Comparative analysis identified 28 unique GS and 3 unique WB protein coding genes, and the variable surface protein (VSP) repertoires of the two isolates are completely different. The promoters of several enzymes involved in the synthesis of the cyst-wall lack binding sites for encystation-specific transcription factors in GS. Several synteny-breaks were detected and verified. The tetraploid GS genome shows higher levels of overall allelic sequence polymorphism (0.5 versus <0.01% in WB). The genomic differences between WB and GS may explain some of the observed biological and clinical differences between the two isolates, and it suggests that assemblage A and B *Giardia* can be two different species.

## Author Summary

*Giardia intestinalis* is a major contributor to the enormous burden of diarrheal diseases with 250 million symptomatic infections per year, and it is part of the WHO neglected disease initiative. Nonetheless, there is poor insight into how *Giardia* causes disease; it is not invasive, secretes no known toxin and both the duration and symptoms of giardiasis are highly variable. Currently, there are seven defined variants (assemblages) of *G. intestinalis*, with only assemblages A and B being known to infect humans. Although assemblage B is the most prevalent worldwide, it is inconclusive whether the various genotypes are associated with different disease outcomes. We have used the 454 sequencing technology to sequence the first assemblage B isolate, and the genome was compared to the earlier sequenced assemblage A isolate. Large genetic differences were detected in genes involved in survival of the parasite during infections. The genomic differences between assemblage A and B can explain some of the observed biological and clinical differences between the two assemblages. Our data suggest that assemblage A and B *Giardia* can be two different species. The identification of genomic differences between assemblages is indeed very important for further studies of the disease and in the development of new methods for diagnosis and treatment of giardiasis.

## Introduction

*Giardia intestinalis* (syn *G. duodenalis* and *G. lamblia*) is a major contributor to the enormous burden of diarrheal diseases, as causes of morbidity and mortality worldwide. The human prevalence rates range from 2–7% in developed countries to 20–30% in most developing countries [1]. Infection of young farm animals is a major economical problem and *G. intestinalis* is a potentially zoonotic organism [2]. Nonetheless, the mechanism of giardial disease is poorly understood [3]. It is not invasive and secretes no known toxin and there is no general consensus on the cause of symptoms. However, recent data suggest that there is induction of apoptosis in intestinal epithelial cells during acute human giardiasis and that diarrhea is partly a result of increased intestinal permeability due to the apoptosis [4]. Chronic infections are common and in a hyperendemic area, 98% of drug-cured children are reinfected within six months [5]. On the other hand, about half of the infections are asymptomatic and frequently the infection spontaneously resolves [3]. Thus, both the duration and symptoms of giardiasis are highly variable.

Currently, there are seven defined genotypes (assemblages) of *G. intestinalis* with only assemblages A and B being known to infect humans. Although assemblage B is the most prevalent worldwide [2], it is inconclusive whether the various genotypes are associated with different disease outcomes [6],[7],[8]. Difficulties with growth of *Giardia in vitro* and the tetraploid genome [9] divided between two nuclei, have precluded the efficient use of biochemical, genetic and molecular biology approaches to experimentally correlate genotypic differences with virulence. Only two *Giardia* isolates (WB-assemblage A and GS-assemblage B) have been successfully cultured and studied in any detail at the molecular level *in vitro* [1]. Early studies suggested large sequence differences between the genes of WB and GS since the nucleotide sequence in the coding region of the triose phosphate isomerase (tpi) gene showed only 81% identity between the WB and GS isolates and the non-coding regions were too different to be aligned [10]. Genetic differences between WB and GS have been confirmed in several other genes in more recent studies [11],[12],[13]. Several biological differences have also been identified between the WB and the GS isolates [14] and GS is currently the only *Giardia* isolate that has been used successfully in experimental infections in humans [15] and adult mice [16]. It has even been suggested that assemblage A and B parasites should be considered as two different *Giardia* species [17].

Genome sequencing and comparative genomics can be used to identify genetic characteristics that are either unique or shared by all *G. intestinalis* assemblages and this approach has been used successfully for other protozoan parasites (e.g. *Plasmodium* and *Trypanosomatids* [18],[19]). The genome sequence of *Giardia* WB was recently published and it was shown to have a highly streamlined genome [20]. In order to understand in greater details the differences between *Giardia* assemblage A and B we decided to produce a draft genome sequence of the GS isolate. We chose to use the 454 sequencing technology (Roche) to characterize the genome of GS, due to the rapidness of data generation and a read length long enough to enable *de novo* assembly of sequence reads. Since its launch in 2005, the 454 technology has [21] been successfully used in a number of genome sequencing projects, most notably in the resequencing of the human genome [22], the sequencing of a Neanderthal mitochondrial genome [23] and several bacterial species [24]. However, to our knowledge this is the first study to use the 454 technology to sequence the genome of a protozoan parasite.

We have produced a draft genome sequence of the assemblage B isolate GS, using a combination of *de novo* sequencing and resequencing, and compared it to the genome of WB. Our findings show only a few assemblage-specific genes, except for the Variable Surface Protein (VSP) gene family where the repertoires of the two isolates are completely different. This study has improved the annotation of the WB *Giardia* genome and provided a framework for further experimental investigations of clinical and biological differences between assemblage A and B *Giardia* isolates.

## Results

### General features of the *G. intestinalis* GS genome

The published *G. intestinalis* WB genome is 11.7 Mbp in size, distributed in 306 contigs on 92 scaffolds [20]. Originally, 6470 open reading frames (ORFs) were identified in the WB genome but only 4,787 were shown to be associated with transcription [20]. This is slightly less than the number of protein coding sequences found in the yeast *Saccharomyces cerevisiae* [25] but more than the number of coding sequences found in the intestinal, eukaryotic microbial pathogens *Encephalitozoon cuniculi* and *Cryptosporidium parvum* [26],[27]. Three rounds of sequencing of the GS genome using the 454 FLX sequencer generated 808,181 high-quality reads with an average length of 227 bp (182 Mbp; 16× coverage). The final assembled sequence was distributed in 2,931 contigs with an average length of 3,753 bp (Table S1). Automated ORF prediction identified 6,768 ORFs and manual curation of the data (see Methods) resulted in a final set of 4,470 intact (mean length 1,836 bp) and 221 interrupted protein coding genes. The mean intergenic distance was 130 bp. The number of protein coding genes is similar to what was observed in the WB genome and the intergenic distance is smaller due to annotation of an additional 754 open reading frames that were never annotated in the pioneering genome project. A small number of genes (64) were fragmented and in these cases specific primers were designed and the genes were amplified by PCR and sequenced. Fifty-eight of the fragmented genes were found to be disrupted by frame-shifts caused by single-base indels due to 454 sequencing errors and only 6 genes had actual frame-shifts (Table S2). Approximately 75% of the assembly is annotated as coding. However, the coding content is 90% in contigs with two or more genes annotated. The two genomes showed 77%±5% nucleotide and 78%±14% amino-acid identity in protein coding regions. The average GC-content was 46.5% in coding regions and 37.8% in intergenic regions. The codon usage was similar in the two isolates, but the codons in the GS ORFs have a higher level of A/T in the third positions compared to WB. We identified 124 small RNA genes in the GS genome, including tRNAs (Table S3 and Text S1). The GS isolate contains 69 tRNAs of the same number of isotypes (45) as the WB genome (Text S1). Most tRNAs are encoded by one gene, but the tRNA^Gln(TGG)^ gene, containing an intron, was found in 7 copies plus one copy without the intron.

### Unique genes in WB and GS

It has been suggested that the WB and GS isolates belong to different *Giardia* species [17] so we decided to study the protein coding capacities of the two isolates in order to address this issue. Comparisons between the sets of predicted protein coding genes showed that 673 WB genes lacked significant sequence similarity to any of the predicted GS genes. Searches at the nucleotide level identified conserved ORFs in GS corresponding to orthologs of 80 of the WB protein coding genes and these were therefore subsequently added to the GS annotation. Five WB protein coding genes showed sequence similarity to chromosomal GS regions without any corresponding full-length ORFs, which indicated the presence of pseudogenes in GS. Of the remaining 588 genes that lacked GS orthologs, 585 coded for proteins shorter than 200 amino-acids (aa) and lacked similarity to sequences present in the public databases. This suggests that these predicted proteins are most likely erroneous annotations, rather than unique WB proteins. Thus, surprisingly, only three WB genes coding for proteins longer than 200 aa are completely absent from the GS genome, all of which code for hypothetical proteins (Table 1). However, it should be noted that for this analysis members of large *Giardia*-specific gene families, such as the VSPs, the ankyrin-repeat domain containing Protein 21.1, High Cysteine Membrane Proteins (HCMPs) and NIMA-Related Kinases (NEKs) were excluded.

**Table 1:** Unique genes in GS and WB.

Gene	Isolate	Gene family^1^	length/aa	Contig	E-value^2^	Annotation^2^	Organism^2^	Tree^3^
GL50803_3386	WB		528	AACB02000002^5^				
GL50803_4447	WB		259	AACB02000014^5^				
GL50803_101423	WB		248	AACB02000001^5^				
GL50581_2613	GS		270	2512	6E-69	Beta-lactamase domain protein	*Desulfatibacillum alkenivorans* AK-01	S1A
GL50581_2037	GS		354	2425	4E-26	Conserved hypothetical protein	*Clostridium perfringens* B str. ATCC 3626	S1B
GL50581_2038	GS	A	230	2425				
GL50581_2039	GS	A	158	2425				
GL50581_2040	GS	B	198	2425				
GL50581_2041	GS		138	2425				
GL50581_2042	GS		663	2425				
GL50581_3192	GS		257	2785	1E-88	Conserved hypothetical protein	*Clostridium ramosum* DSM 1402	S1C
GL50581_3340	GS	C	307	3001	2E-66	Putative replication-accociated protein REP1	*Giardia intestinalis* BRIS/92/HEPU/1541^4^	S1D
GL50581_3339	GS	D	244	3001				
GL50581_3637	GS		1936	3065				
GL50581_1168	GS		250	1703				
GL50581_1169	GS		355	1703				
GL50581_1170	GS		274	1703				
GL50581_1171	GS		311	1703				
GL50581_1633	GS	D	244	2481				
GL50581_1632	GS	C	306	2481	4E-69	Putative replication-accociated protein REP1	*Giardia intestinalis* BRIS/92/HEPU/1541^4^	S1D
GL50581_4567	GS		721	2481				
GL50581_3038	GS	A	252	2663				
GL50581_3039	GS	C	392	2663	2E-67	Putative replication-accociated protein REP1	*Giardia intestinalis* BRIS/92/HEPU/1541^4^	S1D
GL50581_3319	GS	A	222	2914				
GL50581_180	GS	B	182	2914				
GL50581_3321	GS		150	2921	7E-42	Hypothetical protein MS1399	*Mannheimia succiniciproducens* MBEL55E	1B
GL50581_3333	GS	C	335	2936	2E-18	Master replication protein	Faba bean necrotic yellows virus (isolate SV292-88)	S1D
GL50581_3342	GS		304	3020				
GL50581_7	GS		305	38				
GL50581_62	GS		245	42				
GL50581_100	GS	D	237	120				

1 Indicates internal sequence similarity within the set of unique proteins.

2 The best matches in BLAST searches against the non-redundant protein database at NCBI.

3 Maximum likelihood phylogenetic trees found in the indicated figures.

4 The best non-*Giardia* matches are against viral sequences.

5 Contig names and from GiardiaDB, release 1.1 (http://www.giardiadb.org/giardiadb/).

For 754 of the predicted GS genes no annotated orthologs could be detected in the WB genome (www.giardiadb.org). However, searches against the WB scaffolds revealed that these have conserved ORFs, which were not annotated as coding sequences in the WB genome project. An additional four genes corresponded to WB chromosomal regions with putative pseudogenes. The remaining 59 protein coding genes did not show any significant sequence similarity to the WB genome, of these 23 code for proteins longer than 200 aa and are likely coding sequences (Table 1). An additional 4 sequences code for shorter proteins, but are located in proximity to longer unique genes, and a single gene shorter than 200 aa encodes a conserved hypothetical protein. We kept these and annotated them as functional proteins (Table 1). The remaining 31 short proteins without sequence similarity in the WB genome were deemed unlikely to represent functional genes and were therefore not considered further.

**Figure 1:**
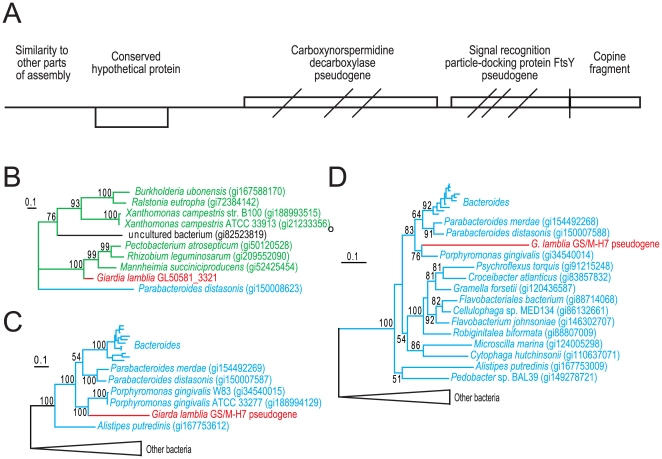
Characterization of a recently introduced chromosomal region in GS. (A) Overview of contig 2921 showing identified genes and pseudogenes. Diagonal bars indicate approximate positions of frameshift mutations in the putative proteins. Vertical bar indicate putative deletion causing loss of the 3′ end of the FtsY gene and 5′ end of a gene coding for Copine-1. Arrows indicate positions of primers used in PCR reactions to connect contig 2921 with contig 2545. Protein maximum likelihood tree based on 125 unambiguously aligned position between the conserved hypothetical protein and all available homologs (B). Giardia sequence is shown in red, proteobacterial sequences in green, and sequences belonging to Bacteroidetes in blue. Protein maximum likelihood trees of carboxynorspermidine decarboxylase (C) and signal recognition particle-docking protein FtsY (D) based on 322 and 297 unambiguously aligned positions, respectively. The Giardia sequences were reconstructed based on alignments of the translation in all three reading frames to functional homologs of the pseudogenes. Only bootstrap support values above 50% are shown.

Thus, 28 protein-coding genes were found to be unique for the GS isolate. Although the majority of these genes code for hypothetical proteins, eight showed sequence similarity to genes present in the public databases. Four of these appear to be of bacterial origin, since the best BLAST matches were to bacterial sequences, and they branched with bacterial genes in phylogenetic trees (Table 1; Fig. 1B; Fig. S1A,B,C). Another four showed similarity to a gene family associated with rolling circle replication and mostly found in viruses (Table 1; Fig. S1D). Homologs to these proteins of putative viral origin have previously been shown to be present in a *G. intestinalis* isolate BRIS/92/HEPU/1541 [28], but they are not present in the WB genome (Table 1). Interestingly, three additional gene families were detected among the unique genes coding for hypothetical proteins, resulting in a total of 19 genes and gene families that were only present in the GS/M-H7 genome (Table 1).

### Recent introduction of a bacterial genomic fragment into the GS/M-H7 genome

To get a more detailed view of the process of gene acquisition in *Giardia* genomes, we examined one short contig in more detail (contig 2921). This contig contained a single unique gene of bacterial origin (GL50581_3321, Fig. 1A). The 3′ end of contig 2921 terminates in a truncated Copine-1 (CPNE1) gene, which suggested that the bacterial fragment has been inserted in the CPNE1 genomic environment (Fig. 1A). PCR and subsequent sequencing demonstrated that the rest of the truncated CPNE1 gene (GL50581_2716) is located in the end of contig 2545, which is syntenic with scaffold CH991778 in the WB genome. Thus, contig 2921 is linked to chromosomal regions showing strong similarity to the WB genome. Searches using the 5′ end of contig 2921 revealed strong similarities to multiple parts of the assembly (Fig. 1A), suggesting that the shortness of the contig is due to assembly errors caused by repetitive sequences.

Searches against protein databases using the sequence outside the annotated conserved hypothetical protein revealed sequence similarity to two bacterial genes coding for carboxynorspermidine decarboxylase and signal recognition particle-docking protein (FtsY), respectively (Fig. 1A). However, there are three frame-shift mutations in the carboxynorspermidine decarboxylase and the *ftsY* genes. Inspection of the assembled reads did not reveal any sequence ambiguities that could explain the apparent frame-shifts in these two genes; resequencing using the Sanger method confirmed this observation. In addition, the 3′ part of the *ftsY* gene is missing from the GS genome, indicating that it is a truncated pseudogene.

Phylogenetic analysis of the intact gene (GL50581_3321) within contig 2921 showed it nested within mostly proteobacterial sequences, most likely indicating a recent bacterial origin (Fig. 1B). Similarly, phylogenetic analyses of the reconstructed putative protein sequences for the two pseudogenes show that the *Giardia* sequences group with *Porphyromonas gingivalis* sequences nested within members of the bacterial Bacteroidetes group (Fig. 1C,D). Indeed, these two genes are found in the same gene order as in the *Porphyromonas* genomes. This strongly suggests a recent transfer to *Giardia* of these two genes in a single event from a close relative of *Porphyromonas*, a genus of bacteria frequently found associated with humans [29]. The relatively long branches leading to the *G. intestinalis* sequences suggest that the pseudogenes have accumulated several mutations in addition to the frameshifts observed (Fig. 1C,D). These observations show that contig 2921 of the *G. intestinalis* GS genome encodes three genes of recent bacterial origin, two of which likely became pseudogenes after the introduction into the genome.

Inspection of the assembly of contig 2921 showed that the average coverage was about half of the expected 16 times coverage. This could be due to random variations in genome coverage but it can also indicate that this region of the genome may not be present in all four copies of the genome in the cell. To test the latter hypothesis we designed PCR primers covering the part of contig 2921 harboring bacterial genes (Fig. 1A). Quantitative PCR analyses showed an approximate copy number of 0.3 for this part of the genome, compared to the single-copy gene beta-giardin (data not shown). This is in agreement with presence of the bacterial gene and pseudogenes in only 1 or 2 of the 4 chromosomal copies in the cell, which provide additional support for a recent introduction into the GS genome.

### Higher frequency of allelic sequence heterozygosity in GS

**Figure 2:**
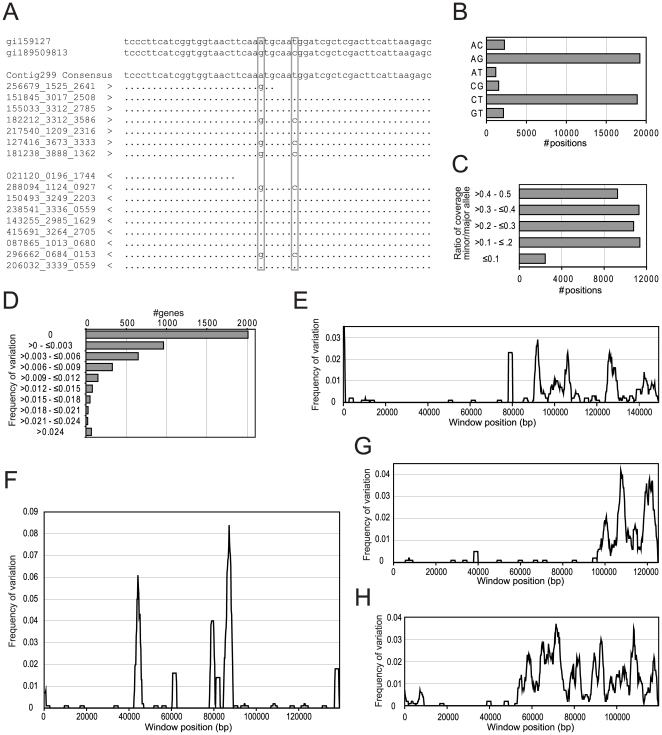
Characterization of sequence variations within GS. (A) Individual sequence reads of a portion of the triosephosphate isomerase (tpi) gene aligned to two previously reported sequences from the GS isolate. (B) Distribution in the six possible two-base combinations for the 45153 variable positions with two alternative bases represented among the individual sequence reads. (C) Analysis of the ratio between the numbers of independent reads for the major and minor base in the positions with two alternative bases. (D) Analysis of the frequency of positions with variations for individual genes. Analysis of the distribution of positions with variations along the four largest contigs in the assembly; contig 2890 (E), contig 2435 (F), contig 540 (G), and contig 1134 (H). Sliding windows of 2000 bp were analyzed in steps of 200 bp.

*Giardia* is a tetraploid organism with two diploid nuclei [9]. Sequence divergence is expected to accumulate in polyploid organisms in the absence of genetic exchange. For example, extensive sequence divergence has been observed between the two former haplotypes in asexual bdelloid rotifers [30]. However, a surprisingly low level of allelic sequence divergence, less than 0.01%, was reported in the WB genome [20]. PCR analyses of genes from patient samples containing assemblage B isolates often show a high degree of sequence divergence in certain positions [2],[31]. These observations could be caused by frequently mixed infections of different assemblage B lineages, or by a higher level of allelic sequence divergence. There are two different haplotypes of *tpi* coding for triose phosphate isomerase in the GS isolate in the public databases. A comparison of these sequences with the sequence reads in the genome show the presence of two distinct classes of sequence reads (Fig. 2A), which strongly suggest allelic sequence variation in the *tpi* gene. This allelic sequence divergence was also verified using PCR amplification and Sanger sequencing of individual clones (data not shown).

A genome-wide analysis of the presence of allelic sequence variation was performed on 8,618,167 positions with 10× coverage or more. A position was defined to contain an allelic sequence variation if two or more independent reads contained an alternative base compared to the consensus. The reads were classified as independent if they started at different positions. Insertion and deletion variation were not included because of the relatively high frequency of such sequencing errors using the 454 technology. Using these criteria, we detected 45,153 positions with two different bases, of which 22,655 were in coding regions and 22,498 were in non-coding regions. A strong bias towards transitions was observed (Fig. 2B). The average coverage for all positions with more than 10 independent reads and positions identified as variable are 16.3 and 18.8, respectively, which shows that the variation was not caused by the collapse of repeated genes in the assembly.

Allowing for a third variant with two or more independent reads detected 106 positions with three different bases. The average coverage for these positions is slightly higher (23.9), which suggests that a fraction of these indeed could represent misassembled duplicated regions. No positions with all four nucleotides represented were found. Thus, the overall level of allelic sequence divergence was 0.53% in the GS genome, compared to less than 0.01% in the WB genome [20]. As expected, the allelic sequence divergence is higher in non-coding than in coding regions, 1.25% and 0.3%, respectively. This high frequency of allelic sequence variations suggests that the “double-peaks” observed in genotyping studies [31] could be explained by allelic sequence variation within a single infecting *Giardia*. The presence of a large number of positions with allelic sequence variation suggests that gene sequences may differ between the two nuclei in the cell. However, analysis of the ratio between the major and minor nucleotide in the variable positions indicate that ratios that deviate from 1∶1 are common (Fig. 2C), which indicates that the two chromosomes within a single nucleus may differ.

Interestingly, the allelic sequence variations were not homogenously distributed among the genes (Fig. 2D). Analyses of the distribution of the allelic variations along the contigs show large regions with very low sequence divergence with high divergence regions scattered within them (Fig. 2E–H). This indicates that a large part of the GS genome is identical between the four copies distributed in the two nuclei, while some parts remain divergent. Thirty-eight percent of the polymorphisms change the protein sequence and these are distributed in 1,962 genes, suggesting that GS has an extended proteome of almost 2000 proteins with a slightly altered primary structure. This can be important for understanding the biology and virulence of the parasite.

### Synteny breaks and super-contigs

**Figure 3:**
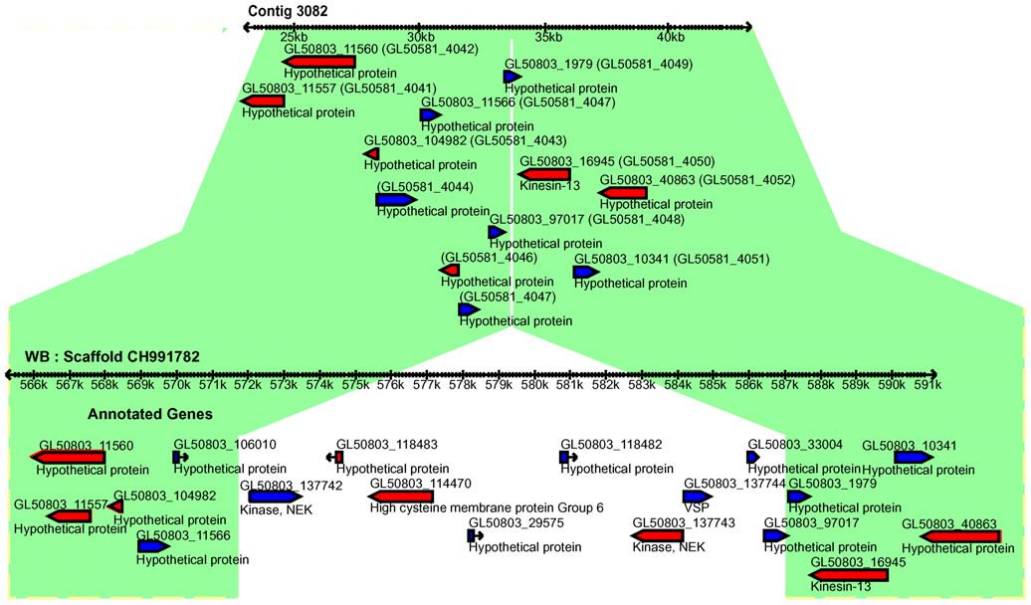
Synteny-break in the GS genome. Comparison of genomic synteny between contig 3082 (upper panel) of the GS assembly and the syntenic region of scaffold CH991782 of the WB genome (lower panel). A 15 kb region is missing from the GS genome. This region contains 7 genes including one VSP, two NEK kinases and one HCMP. Syntenic regions in the two isolates are indicated in green.

Several breaks of genome synteny (41) were identified when the GS contigs were aligned with the WB genome scaffolds (Table S4). Twenty-four of these were verified using PCR and sequencing and 21 of the breaks were between regions in the same WB-scaffold, with 3 occurring between scaffolds. The most common class among the synteny breaks (16 cases) was insertions or deletions of a region in WB or GS. One example of a synteny break in this class is shown in Fig. 3 where a 15 kb region containing 7 genes, among them one VSP, two NEKs and one HCMP are missing in GS. Indeed, VSPs, NEKs, HCMPs and also protein 21.1 were often found associated with insertions or deletions (12 cases). Additionally, VSP fragments were detected at contig edges in GS where the syntenic region in WB is devoid of such proteins. These two observations suggest that VSPs are not confined to certain genomic locations. Another interesting observation is that regions missing from GS but present in WB had a higher GC-content than the average for the genome (Fig. S2). The GC%-profiles of WB scaffolds reveal that the genomic regions where VSPs localize have a higher GC content than the surrounding genome and that these high GC% islands may be important for VSP regulation.

Intra-scaffold rearrangements were the second most prominent category of synteny breaks with 8 detected events. Of these, we detected a recombination event between two dipeptidyl-peptidase I precursors (GL50803_28651 and GL50803_22553) that creates “hybrid proteases”. However, we could also detect non-recombinant variants by PCR analyses indicating that there are two different variants in the GS genome; one similar to WB and one due to recombination between the two protease genes.

A fraction of putative recombination events could not be confirmed by PCR amplification and Sanger sequencing, indicating that these genes were incorrectly assembled. Mis-assembly occurred between genes in gene families containing highly conserved nucleotide stretches such as histones, protein disulfide isomerases, peroxiredoxins and acyl-CoA synthetases. This likely reflects a limitation due to the relatively short read-length obtained by 454 FLX sequencing (250 bp) compared to traditional Sanger sequencing.

The draft GS genome sequence is highly fragmented with 2,931 contigs of an average length of 3,753 bp. In order to see if it is feasible to generate larger contigs we designed primers against truncated genes and contig ends that were predicted to be close according to the synteny analysis. Twelve super-contigs of total size 1,363,697 bp, (corresponding to around 10% of the total genome) were produced after running 28 PCR reactions followed by Sanger sequencing of the products (Table S5). This shows that synteny analysis is useful for the generation of super-contigs and importantly that it is technically feasible to close the sequence gaps.

### Promoters

**Figure 4:**
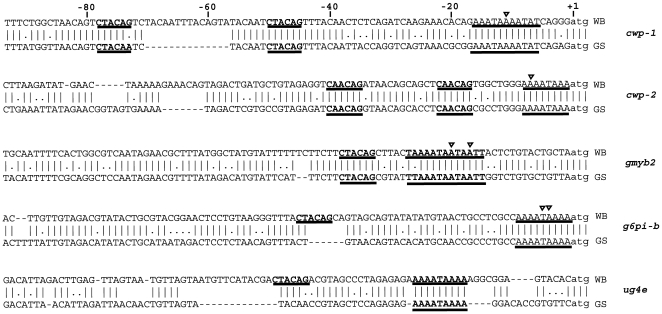
Comparison of encystation-specific promoters. The promoters of the cyst-wall proteins-1 and 2 (CWP-1 and CWP-2), the encystation-specific transcription factor (gMyb2), the key enzymes involved in the synthesis of cyst wall sugars, glucosamine-6 phosphate isomerase (G6PI-B) and UDP-glucosamine-4 epimerase (UG4E) were aligned using the program CLUSTALW. Poly A rich initiator regions and gMyb2 binding-sites are underlined. Note that the G6PI-B and UG4E promoters are missing the gMyb2 binding-sites, whereas they are found in the corresponding positions in the promoters of CWP-1 and –2 and gMyb2.

Promoters in *Giardia* are short (around 50 bp) and the main feature is an initiator-like AT-rich sequence around the ATG start codon, which is enough to drive transcription [32]. AT-rich stretches around the ATG start codon could be found in most GS genes (data not shown), but apart from these, there is very little intergenic sequence conservation, consistent with earlier observations of a few GS genes. Encystation-specific promoters in WB are also short, with 65 bp found to be sufficient for a developmentally regulated promoter [33]. An alignment of the promoters (−100 to +3) from the three major cyst-wall proteins CWP 1–3 from WB with the orthologs from GS showed a high degree of conservation in the 65 bp directly upstream of the start codon (see alignment of CWP 1 and 2, Fig. 4). The transcription factor Myb2 has been shown to bind to the CWP promoters and its own promoter [34]. The GS Myb2 protein is well conserved (77% amino acid identity) and so is the 65 bp directly upstream of the ATG start-codon, including the Myb2 binding site (Fig. 4). This suggests that the cyst-wall proteins and Myb2 protein are regulated in the same way during encystation in the two isolates. The key-regulatory enzyme in WB, glucosamine-6 phosphate isomerase, has a promoter that is similar to the cyst-wall promoters [33]. However, the promoter of this enzyme in GS is not similar to the promoters of the cyst wall proteins (Fig. 4) and, most importantly, it lacks a typical Myb2 binding sequence. The same was found to be true for the last enzyme in the pathway, UDP-*N*-acetylglucosamine 4′ epimerase [35] (Fig. 4). The GS isolate is known for its poor encysting ability *in vitro* [36] and may suggest that the regulation of cyst-wall sugar synthesis during early encystation is different in GS.

### Introns and splicing

Four mRNA introns were identified in the WB genome after combining cDNA and genome sequences [20]. The introns are variable in length (32 to 220 bp) with conserved 5′ and 3′ motifs. Introns of similar sizes were found in the corresponding genes in GS and the 5′ (consensus **G**/C **TAT GT** ) and 3′ motifs (consensus A/C CT **A**/G **A**C **A**/C C**A**C**A**G) were conserved, whereas the sequences in the rest of the introns were highly diverged. Interestingly, we found three unique contigs containing the intron corresponding to the intron of ORF 35332 in WB. The longest contig (c2890) has a consensus 3′motif, while the two other contigs (c299 and c305) have an A to G mutation in the branchpoint A that is crucial for splicing. Thus, these are three allelic variants of the intron and potentially pseudo-introns. Putative spliceosomal RNAs were recently predicted in the WB genome [37]. Only the U4 and U5 RNAs showed high sequence identity (92 and 85%, respectively) with GS, whereas the U1, U2 and U6 RNAs showed less than 80% identity (Table S3). This is surprising, considering that all other verified small RNAs showed a high level of sequence identity and suggesting that some of the predicted U RNAs are not true spliceosomal RNAs or that there are less constrains in the nucleotide sequence of the giardial spliceosomal RNAs.

### Gene families: Variant-specific surface proteins (VSPs)

Earlier studies using Southern blot analyses suggested that the GS genome contains approximately 150 VSP genes [38]. A search of all the reads from the GS data set using TBLASTN with the conserved C-terminal VSP region gave 3,183 hits with more than 80% identity over 30 aa. With a sequence coverage of 16× we could estimate that 200 VSP genes are present in the GS genome. In our study only 16 complete VSP genes were obtained and most of them were short. The low number of identified full-length VSP genes is most likely due to assembly problems caused by the repetitive nature of these genes. The higher levels of allelic sequence divergence in GS most likely also caused problems in the assembly of the VSP genes. We used the 16 ORFs coding for complete GS VSP genes and 188 VSP genes in WB to search for VSP genes in the GS genome. In this search, 15,249 reads over 100 nt were identified, corresponding to 1.9% of all reads. None of these GS VSPs showed a high degree of similarity to VSPs from WB (30–70%, average 55%), except in the conserved CXXC motifs and the C-terminal region. This is in agreement with earlier studies that have suggested that the two isolates have unique VSP repertoires [38],[39]. It is clear that more data using other sequencing platforms is needed to get a view of the complete VSP repertoire in GS.

### Gene families: Kinases, HCMPs and alpha-giardins

The largest gene family in the WB genome was the kinases with 276 putative members [20]. No histidine- or tyrosine-specific kinases were identified and only four giardial kinases contain membrane-spanning regions. The core kinome in *Giardia* is the smallest among eukaryotes thus far with more than 70% of the kinases in WB belonging to the NEK kinase group [20]. We found that certain NEK kinases were highly conserved between the two isolates, whereas others were highly diverged or missing. We identified 360 ankyrin motif-containing proteins in GS (Table S6), with the number of repeats/protein ranging from 1–28. The ankyrin-repeats are commonly localized downstream of the kinase domain in NEKs or in multiple repeats in the 21.1 protein family, indicating that this is an important protein/protein interaction domain in *Giardia*.

The HCMP gene family [40] was discovered during the analysis of the WB genome and is similar to the VSP gene family, except for the absence of the conserved C-terminal CRGKA sequence. Twenty-one full-length HCMPs were identified in GS (Table S6) and many were not complete due to assembly problems. Similar to the NEK gene family, some of the HCMP proteins were highly conserved between WB and GS, while others are highly diverged or absent.

Alpha-giardins is a cytoskeleton gene family that is unique to *Giardia* but it is related to annexins [41]. All the 21 alpha-giardin genes in WB were conserved in GS along with the genome synteny. The amino acid identity of the alpha-giardins between the two genomes is between 70 to 95%, with the exception of alpha-7 giardin, which only displays a 57% identity. Alpha-2 giardin was recently proposed to be assemblage A specific [42]. We found an alpha-2 giardin-like gene in GS with 92% aa identity in a syntenic position but the alpha-1 giardin was less conserved with 87% aa identity and most of the changes were found in the N-terminal region.

### Major cellular processes in *Giardia*

*Giardia* is a micro-aerophilic intestinal parasite with a very limited metabolic repertoire [43], containing no classical mitochondrion, no Krebs cycle or nucleotide and amino acid synthesis genes or enzymes required for *de novo* synthesis of lipids [20]. Many key metabolic enzymes are bacterial-like, including the arginine metabolic pathway that is used for energy production in trophozoites [43]. Phylogenetic analyses indicate that these genes were acquired by the diplomonad lineage via lateral gene transfers from bacteria relatively recently, rather than being retained from a bacterial-like eukaryotic ancestor [20],[44],[45],[46]. We found no differences in the metabolic gene content between the WB and GS isolates. There are 149 genes in the WB genome that have been identified as good drug targets [20] as defined by Hopkins and Groom [47], all these genes are also found in the GS genome. This suggests that drugs directed against these particular genes can have an effect on parasites from both assemblages.

**Figure 5:**
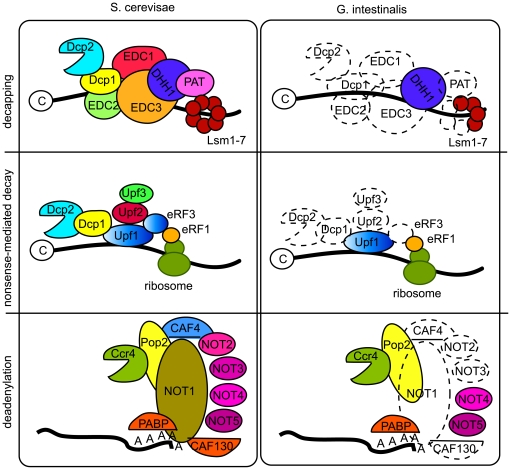
mRNA degradation pathways in Giardia and yeast. The major mRNA degradation pathways in Giardia compared to the corresponding pathways in yeast. Decapping: Decapping in S. cerevisiae involves the decapping enzymes Dcp1–2, EDC1–3, DHH1, PAT and Lsm1-7. In Giardia only DHH1 and three Lsm proteins were identified. Nonsense-mediated decay: The process involves Dcp1-2, Upf1-3 and eRF1 and 3 in yeast. Giardia has only orthologs to Upf1 and eRF1. Deadenylation: Giardia lacks the PARN and Pan2/Pan3 systems. In the CCR4-NOT complex only Ccr4, Pop2, NOT4 and 5 and PABP are found.

One of the main observations of the WB genome project was the simplicity of major cellular machineries [20]. We investigated the same cellular processes studied in detail in the WB genome (e.g. DNA replication, transcription, polyadenylation and actin cytoskeleton) [20], and came to the same conclusion for the GS genome, i. e. *Giardia* has a minimal and simplified cellular composition. We also extended these analyses to several more basic cellular processes and we found the same pattern. One example is the rudimentary composition of the mRNA degradation system (Fig. 5). No decapping enzymes are present, but two typical 5′ to 3′ exoribonucleases were detected. The PARN and Pan 2–3 deadenylation complexes were not detected, but weak homologs for a few proteins in the CCR4-Not complex were identified (Fig. 5). The catalytically important proteins Rpr-4 and -40 of the exosome complex were identified, but only Rpr-45 of the ring structure. It was recently shown [48] that nonsense-mediated decay of mRNAs is present in *Giardia* but at lower efficiency. The Upf-1 protein of the nonsense-mediated decay machinery was identified in both genomes, but Upf-2 and –3 and the other proteins found to be important for nonsense mediated decay in yeast and humans were not (Fig. 5), which may explain the low efficiency of the process in *Giardia*. It was recently shown that the Upf-1 protein is an important regulator of the stability of the cyst-wall protein transcripts during encystations [48] and it will be informative to see how the nonsense mediated decay machinery is composed in *Giardia*.

An analysis of the WB genome showed no evidence of true myosin genes [20], suggesting that cytokinesis is not performed by an actomyosin ring in *Giardia*. However, homologs to actin and the mitotic cyclins A and B were detected in both *Giardia* isolates. Furthermore, we identified giardial homologs to several Mitotic Exit Network (MEN) proteins; Tem1, Cdc5, Cdc14, Cdc15, Bub2 and Mob1 as well as two members of the related FEAR complex; the kinase Cdc5 and the protease Esp1 (see Fig. S3). Our results suggest that the regulation of cytokinesis in *Giardia* is similar to the process in other eukaryotes, even if no strong myosin ortholog has been identified [20].

## Discussion

In this study, we have produced a draft genome sequence of the assemblage B *Giardia* isolate GS. The sequence has numerous gaps, which makes it less complete than the published genome of *Giardia* WB. Given that approximately 12–13% of the WB genome is composed of repeated gene families dispersed over the genome, and that such regions are difficult to assemble with short reads, it is likely that most of these gaps represent repeat regions. This is also supported by comparing the genomic synteny of the two data sets. We found that most contig ends had an interrupted gene sequence belonging to one of the large gene families in *Giardia* (Protein 21.1, NEKs, VSPs or HCMPs). However, our strategy has resulted in reasonably long contigs that provide sufficient information for extraction of the core gene content. Our proof of concept for this shows that we indeed identified orthologs to all annotated protein-coding genes from non-repeated WB gene families in the GS assembly, except 3 (Table 1). This study further supports the concept that for most purposes, a “quick and dirty” approach is sufficient for comparative genomics to be highly informative [49]. This type of strategy is particularly useful for resequencing closely related strains and isolates where a high quality reference sequence is available. Using this approach, we identified several genetic and genomic differences between the GS isolate and the previously sequenced assemblage A isolate WB: (1) unique, isolate-specific proteins, (2) unique patterns of allelic sequence divergence, (3) differences in genome synteny, (4) differences in the promoter regions of encystation-specific genes and (5) differences in the VSP repertoires. These multiple variations may help explain many of the known biological differences between the GS and WB isolates.

### Unique proteins

Lateral gene transfer is increasingly appreciated as an evolutionary mechanism in microbial eukaryotes [50],[51], and metabolic adaptations via gene acquisitions have been shown to occur in diplomonads on longer evolutionary timescales [20],[44],[45],[46]. The comparative study identified 3 unique WB and 28 unique GS proteins, suggesting that gene loss or gain is ongoing within *Giardia*. This is in agreement with earlier findings, although the rate of the process appears relatively low. Although most unique proteins are hypothetical, there are also examples of recently introduced bacterial genes in the GS genome. One example of this is a protein coding gene flanked by two bacterial pseudogenes, indicating a very recent introduction of a member from the Bacteroidetes group. To our knowledge, this is the first report of a “dead-on-arrival” prokaryotic pseudogene incorporated into a eukaryotic nucleus. Until now no isolate-specific genes have been identified in *Giardia*, and further studies will show what functions these genes have in the parasite. These unique genes may be of importance for the development of new tools for diagnosis and typing of *Giardia*. If strain-specific genes from each assemblage are expressed at relatively high levels in trophozoites and cysts in all isolates, it may be feasible to develop antibody-based genotyping/detection assays. The role of these unique genes during host-parasite interactions will also be of great interest for future studies.

### Allelic sequence divergence

One surprising result from the WB genome project was the low level of allelic sequence divergence (<0.01%) [20]. We detected a dramatically higher level of allelic sequence divergence in the GS isolate (average 0.5%). Our analyses indicate that the sequence divergence between haplotypes for most genes is much smaller than the divergence between the genes from the two isolates (Fig. 2D). This contradicts a recent report where genes classified into both assemblage A and B were found in the same *Giardia* isolate [11]. In that study, the GS isolate was shown to contain actin genes from both assemblages and certain intergenic regions showed >99% identity to the corresponding region in WB. We failed to identify any of these reported assemblage A-type GS sequences [11] in our 16× coverage whole genome shotgun sequencing dataset. Unfortunately, the level of genetic exchange between *Giardia* assemblages cannot be determined until more genomic datasets from *Giardia* isolates become available.

The observed allelic variations are likely the result of interactions between mechanisms that create and reduce variation between the four copies of the genome in the cell. The major sources of variations are probably mutations and DNA recombination, which has been proposed to occur between different *Giardia* isolates [52]. It is not likely that the mutations have been induced by genetic drift during asexual mitotic growth *in vitro* since this is the original clone isolated by Nash et al. [17] and it has been grown relatively few generations *in vitro*. There are also other mechanisms that could change the level of genetic variation between the four copies of the genome. Diplomixis, a recombination process between *Giardia*'s two nuclei, shown to occur during encystation [53], could be an important mechanism. This is a unique process for *Giardia* with its two nuclei and genes related to meiotic processes in other organisms were suggested to be important in this process [53],[54]. All identified meiosis-related genes identified in WB [55],[56] can be found in GS. Several are well conserved (Spo11–78% aaID, Dmc1–92% aaID, Msh6–78% aaID), others are not as conserved (Mre11–62%, Rad50–60%) and some even show deletions (15 aa in Rad52) or insertions (14 aa in Mlh1) in important regions. The higher levels of allelic sequence divergence in GS could suggest that diplomixis is less efficient in the GS isolate compared to the WB isolate, although further investigations are needed to separate the effects of different mechanisms creating and reducing allelic variation in *Giardia* isolates.

### Synteny breaks

We identified and confirmed several breaks of gene synteny when the GS contigs were aligned with the WB genome scaffolds. Twenty one of the synteny breaks occur between regions in the same WB-scaffold with 3 occurring between different WB scaffolds. The most common class of synteny breaks was insertions or deletions of chromosomal regions containing members of the large *Giardia*-specific gene families VSPs, NEKs, HCMPs and Protein 21.1. In at least two cases we detected two different variants of gene synteny; one identical to WB and one unique for GS. These recombination events occurred between two very similar genes localized within 15 to 30 kbp of each other. It is possible that these kinds of inversions between similar regions are more common than what we have detected here and it is also possible that there are differences between the two nuclei.

### Encystation-specific promoters

Several characteristics of *Giardia* influence the epidemiology of human giardiasis: (a) the rate of trophozoite growth; (b) the encystation efficiency; (c) the size of the infective dose; (d) the excystation efficiency; (e) the viability of the secreted cysts and (f) the number of hosts, since zoonotic parasites have larger reservoirs. Most assemblage A parasites grow faster and differentiate (encyst and excyst) better *in vitro* than the few studied assemblage B isolates [9],[36],[57],[58],[59]. We found that Myb2 binding sites in the promoters of two enzymes involved in production of cyst-wall sugars in WB were missing in GS. This can potentially explain the poor encystation observed in GS, and furthermore suggests that the encystation stimuli may be different between assemblage A and B parasites. In order to further understand the epidemiology of giardiasis, it will be important to determine if there is a correlation between *Giardia* assemblages and cyst production in either infected humans or animals.

### VSP repertoires

The most well characterized virulence factors in *Giardia* are the VSP proteins [14],[59]. The genome sequencing of WB found approximately 200 genes encoding different VSPs spread over the 5 chromosomes [20]. Our results showed that the VSP repertoires are very different in GS compared to WB. Certain VSPs are known to have toxin-like motifs [60] and it is possible that the differences in symptoms seen during *Giardia* infections are not due to assemblage differences, but rather because of differences in VSP repertoires and expression.

### Are humans infected by two different *Giardia* species?

One major issue in the *Giardia* research field has been the identification of genetic differences between the two human-associated *Giardia* assemblages A and B that would explain the observed phenotypic differences. Early genetic studies suggested that the levels of genetic diversity between the assemblage A and B parasites parasites are sufficient to recognize them as different species [17],[61]. It is difficult to define a general species concept in eukaryotic microbes and there are more than a dozen of alternate eukaryote-specific “species concepts” used currently [62]. The biological species concept emphasizes the property of reproductive isolation but it is not applicable for organisms that multiply far more often by asexual than sexual reproduction [62]. The ecological species concept emphasizes occupation of a distinct niche or adaptive zone whereas one version of the phylogenetic species concept emphasizes diagnosability and another version requires monophyly of members of the species in phylogenetic trees [63],[64],[65]. The WB and GS isolates can be considered as separate species according to the phylogenetic species concept because they group into difference assemblages in genotyping studies and our data show an extensive primary sequence divergence across the majority of the genes. However, not enough data is available to define them as separate species according to many of the other species concepts, e.g. they both infect humans. Nevertheless, several biological differences have been detected between WB and GS and/or assemblage A and B isolates. WB is more easily stably transfected by episomal plasmids than GS [66]. Cytogenetic studies showed that certain assemblage A and B isolates differ in the number of chromosomes in each nucleus [67] and pulse-field analysis detected differences in chromosome size [68]. The repertoire of VSP proteins is very different in the two different isolates [68]. The GS isolate can readily infect mice, whereas the WB is cleared before it can establish an infection [16]. The GS isolate gave more severe symptoms than the assemblage A isolate Isr in experimental human infections [15]. Here we present data that connect phenotypic differences between the WB and GS isolates (poor encystation and no cross-protection) to genetic differences (differences in encystations-specific promoters and VSP repertoire). Our results supports the recent suggestion of a revised *Giardia* taxonomy [69]. However, more data is needed in order to determine if the differences detected between WB and GS is true for all assemblage A and B isolates. This study has provided the tools to do this type of studies, which is important in further studies of giardiasis since the uncertain taxonomy has had a negative effect on the understanding of the disease.

## Materials and Methods

### Reagents and cell culture

Unless otherwise indicated, the reagents were obtained from Sigma Chemical Co, USA. *Giardia intestinalis* strain GS, clone H7 (ATCC50581) trophozoites were grown as described [70]. The GS strain was isolated from a human patient infected in Alaska [17] and H7 is a clone of the original isolate.

### 454 sequencing and assembly

Genomic DNA was extracted from *G. intestinalis* GS trophozoites using the Easy-DNA kit for genomic DNA isolation (Invitrogen, Carlsbad, CA, US, Cat. no. K1800-01). The genomic DNA was sequenced using a Genome Sequencer FLX instrument (Roche). Preparation and sequencing of the sample was performed according to the manufacturer's instructions. Base-calling was performed using the bundled 454 software. The quality of the generated sequence reads was evaluated using the Phred-like [71] quality scores associated with the sequence reads. Only 2.98% of the bases in the pool of sequence reads were shown to have quality values less than 20, which corresponds to 5,445,574 bases. If each one of these bases would have a score equal to 10 this would correspond to a probability of 1 in 10, or 544,557 incorrectly called bases- equal to 0.29% of the total number of sequenced bases. Thus, low quality sequence data is not a problem in the data set.

The 808,181 reads generated from the 454 instrument were clustered using the Newbler sequence assembler from 454 Life Sciences (version 1.1.03) and the reads that were successfully clustered were extracted and reclustered using the MIRA sequence assembler (version 2.9.26×3 for 64 bit Linux) [72]. This combined assembly strategy was required because of the tendency of the Newbler assembler to misinterpret polymorphisms as sequencing errors and introduce artifactual gaps into the sequence. The default parameters were used for both programs. A contamination control of the assembly was performed using BLAST searches against the GenBank non-redundant nucleotide database and contaminating sequences were removed. The clustering and final analysis formed 2,931 contigs over 200 bp with a total assembly size of 11 Mbp (Table S1).

### Prediction of protein coding genes

Gene prediction was performed on the 2,931 contigs using Glimmer version 3.02 [73] and CRITICA [74] using training genes (6,500) from the published *Giardia* genome (isolate WB). The programs have overlapping prediction patterns, therefore duplicated ORFs were removed, as were ORFs without proper start and/or stop codons. The genomic data from this study has been deposited in GenBank with accession number ACGJ00000000 and the genome sequence is reported as recommended by the Genome Standards Consortium [75] (Table S7).

### WB and GS orthologs

Orthologous relationships between putative coding sequences in GS and WB-C6 (ATCC50803) were determined using NCBI BLASTP. The predicted ORFs from GS were queried against a database containing putative coding sequences from WB. The reciprocal best hit was used to identify orthologs. The top hit from each BLAST report was required to have an E-value<10^−10^, amino acid identity above 50% and the high scoring pair had to have a length at least 60% of the CDS length in WB.

### Annotation and curation

The automatic GS annotations were aligned with the corresponding annotations in WB and manually inspected using the Artetmis Comparison tool [76] and SynBrowse [77]. Additional orthologs were identified by examination of the conserved gene order. Conserved, non-overlapping GS ORFs with no annotation in WB were kept in the GS annotation, and their annotations were added to the WB genome. Similarity searches with annotated WB genes with no GS ortholog assignment were used to evaluate differences in genomic gene content. For certain genes, unambiguous ortholog assignments were not possible because of their divergent nature. Truncated genes present at contig ends were listed separately.

### Synteny analysis

Gene synteny was analyzed using SynBrowse and the Artemis Comparison Tool. Synteny information combined with translational BLAST searches provided evidence for orthologous genes located at contig ends. Synteny breaks were verified using PCR. Contigs where PCR did not support a synteny break were split into two separate sequences. Interrupted ORFs were also amplified using PCR and 66 were sequenced using Sanger sequencing. Verification of frame-shifted genes, synteny breaks and joining of contigs were also performed by PCR and Sanger sequencing of the resulting PCR products. Primers were designed manually according to recommendations in the Phusion HotStart polymerase instruction manual (*T_m_* 60°C and 22–25 bp in length) and synthesized by Sigma-Genosys (Text S2). The targets were amplified in a mixture containing 1xPhusion HF buffer with 1.5 mM MgCl_2_ , 200 µM dNTPs, 0.5 µM of the forward and reverse primers, 10 ng GS/M-H7 genomic DNA and 0.8 U Phusion HS DNA polymerase (Finnzymes) in a total volume of 40 µl. The reactions were incubated for 2 min at 98°C followed by (98°C for 15 sec, 55°C for 30 sec, 72°C for 30 sec/1 kb of expected amplicon) ×35 cycles and were subsequently held at 4°C. The PCR products were purified using the QIAquick PCR purification kit according to the manufacturer's recommendations and eluted in 30 µl ddH_2_O. The purified PCR products were sequenced with their respective forward and/or reverse primers at the Uppsala Genome Center using the BigDye® Terminator v3.1 (Applied Biosystems) chemistry followed by capillary electrophoresis on an ABI3730XL sequencer (Applied Biosystems).

### Production of super-contigs

Synteny analysis identified GS contigs that were predicted to be close to but not joined in the assembly. Super-contigs were produced by PCR amplification of the missing regions between GS contigs and the PCR products were sequenced in both directions to obtain paired-read coverage. Primer design, PCR conditions and sequencing were performed as in the synteny analysis section.

### Alignment of ortholog pairs

Pairwise nucleotide and amino acid alignments were created for the ortholog pairs using the blastn and blastp programs in the wublast package and the sequence identity for each alignment were subsequently extracted.

### Codon usage

We used the EMBOSS [78],[79] program cusp to examine codon usage in putative coding sequences in both *Giardia* isolates.

### Analysis of allelic sequence variation

In-house Perl scripts were developed to identify sequence variation in the ace file generated by the MIRA sequence assembler and the 8,618,167 positions in 3,118 assembled contigs with at least 10-fold coverage and lacking indels and ambiguous nucleotides (‘n’) were examined for sequence variations. For a position to be classified as a polymorphism an alternative nucleotide had to be present in at least 2 reads with different start positions.

### Prediction of RNA genes

The prediction of tRNA genes was performed using tRNAScan (version 1.23) [80] with default parameters. Ribosomal and small RNAs were identified by sequence comparison of published RNA genes from *Giardia* WB.

### Phylogenetic analyses

Sets of homologous sequences from the public databases were compiled and aligned using CLUSTALW [81]. Only unambiguously aligned regions identified by manual inspection were used in the phylogenetic analyses. The optimal substitution models for each dataset were determined using MODELGENERATOR, version 0.84 [82] Maximum likelihood analyses were performed using PHYML, version 2.4.5 [83]. In addition, bootstrap analyses with 100 replicates were performed for each dataset with the same parameters.

## Supporting Information

Figure S1 Phylogenetic trees of the identified unique GS genes with homologs in the public databases. Maximum likelihood tree of (A) beta-lactamase (GL50581_2613), two conserved hypothetical proteins: (B) GL50581_2037 and (C) GL50581_3192, and (D) a replication-associated protein (GL50581_3340, GL50581_1632, GL50581_3039 and GL50581_3333).
(0.41 MB PDF)

Figure S2 A sliding-window GC content analysis of a chromosomal region in the WB genome (CH991782) compared to the corresponding region in the GS genome. A custom script was used with a windowsize of 5000 bp and 500 bp steps.
(0.20 MB PDF)

Figure S3 Regulatory cytokinesis proteins in *G. intestinalis* and *S. cerevisae*. White indicates non-identified proteins in *Giardia* and green identified orthologs. A signaling cascade results in the assembly of an actinomyosin ring and cell division.
(0.47 MB PDF)

Table S1 Distribution of contig sizes in the assembled GS genome.
(0.04 MB PDF)

Table S2 Fragmented GS ORFs and results from PCR analyses.
(0.01 MB XLS)

Table S3 Small RNAs identified in *Giardia* GS and WB.
(0.12 MB PDF)

Table S4 Identified synteny breaks in the GS compared to the WB genome.
(0.09 MB PDF)

Table S5 GS supercontigs generated using PCR and Sanger sequencing.
(0.04 MB PDF)

Table S6 Analysis of HCMP and ankyrin repeat proteins in GS compared to WB.
(0.11 MB XLS)

Table S7 Supplementary *Giardia* GS MIGS report.
(0.07 MB PDF)

Text S1 Summary of tRNA genes identified in the GS genome.
(0.06 MB PDF)

Text S2 Oligonucleotides used to connect GS contigs, amplify truncated ORFs and sequence different regions of the genome.
(0.06 MB XLS)
