# Supplementary figures and images for: Draft Genome Sequencing of Giardia intestinalis Assemblage B Isolate GS: Is Human Giardiasis Caused by Two Different Species?
Source: PLoS Pathog. 2009 Aug 21;5(8):e1000560. doi: 10.1371/journal.ppat.1000560 (PMC2723961; doi:10.1371/journal.ppat.1000560)

A

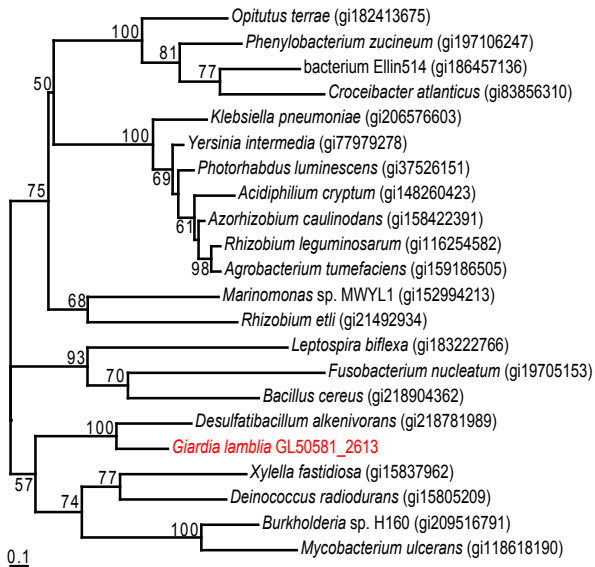

C

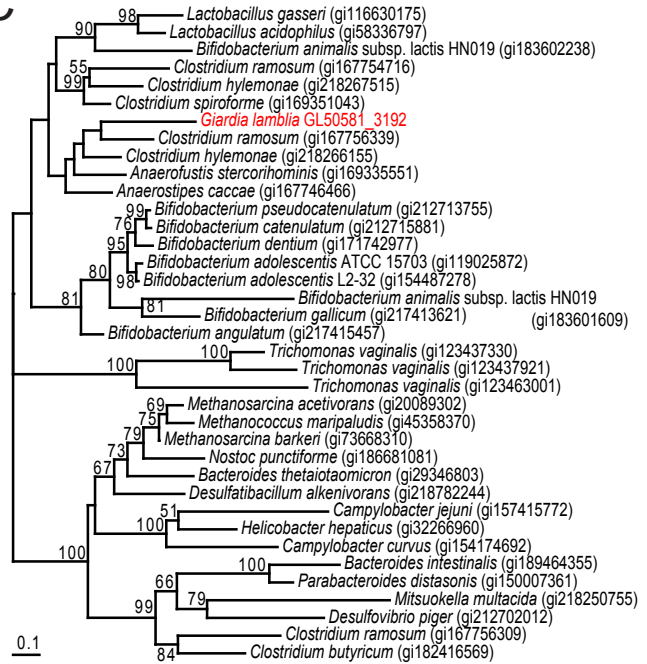

B

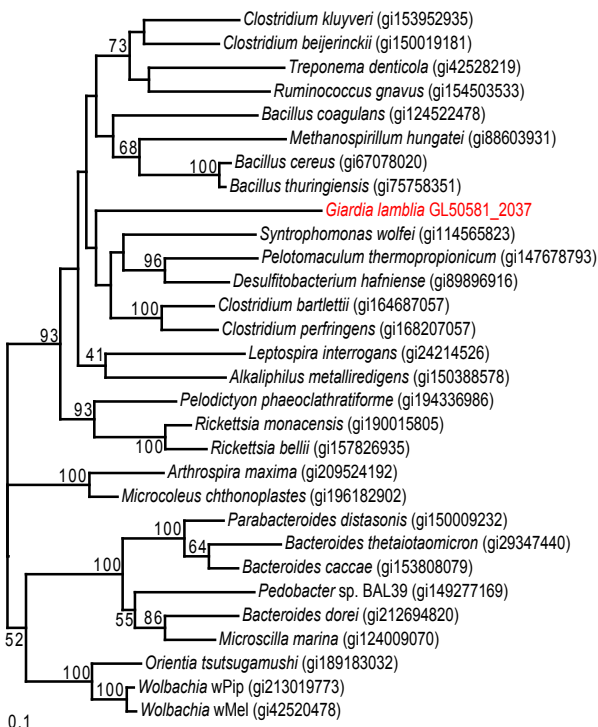

D

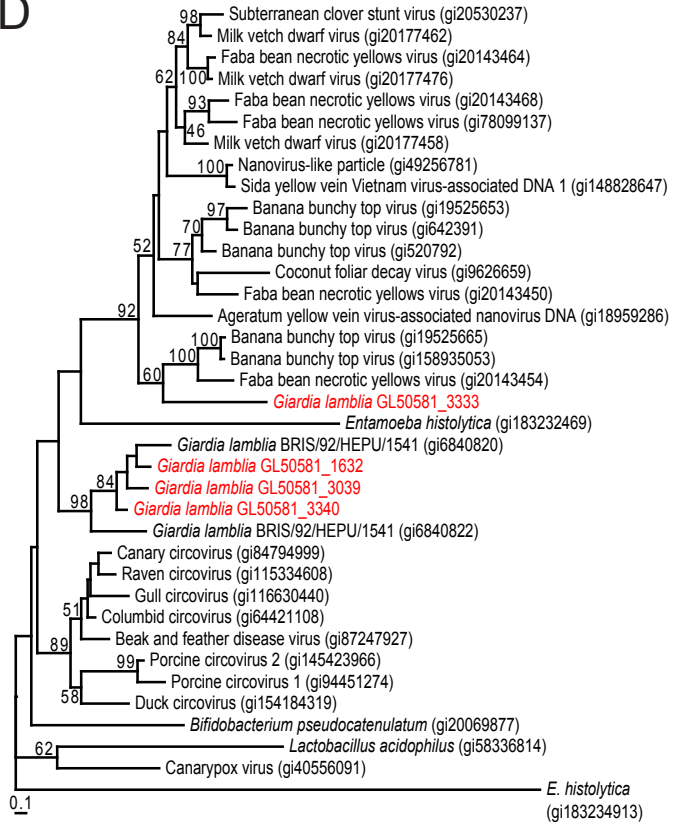

Supplement: Figure S1 — Phylogenetic trees of the identified unique GS genes with homologs in the public databases. Maximum likelihood tree of (A) beta-lactamase (GL50581_2613), two conserved hypothetical proteins: (B) GL50581_2037 and (C) GL50581_3192, and (D) a replication-associated protein (GL50581_3340, GL50581_1632, GL50581_3039 and GL50581_3333). (0.41 MB PDF) [file ppat.1000560.s001.pdf]

Window size: 5000 bp ; Step size: 500 bp

WB-C6 CH991782

GS/M-H7 syntenic contigs

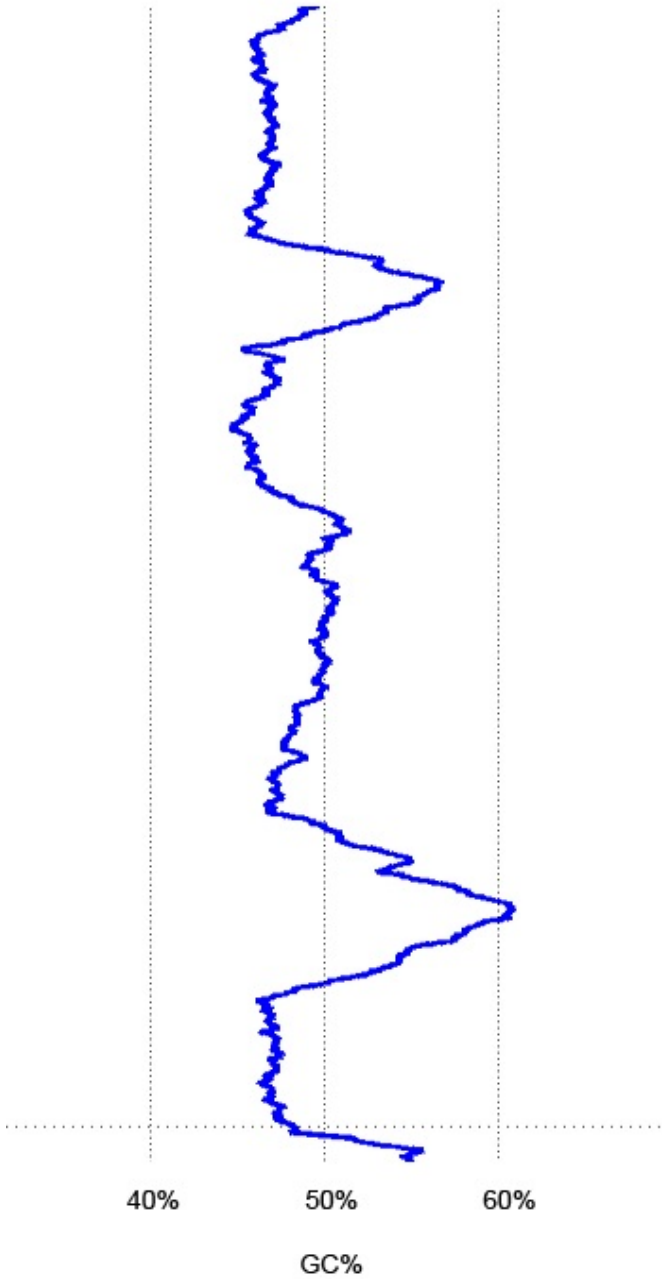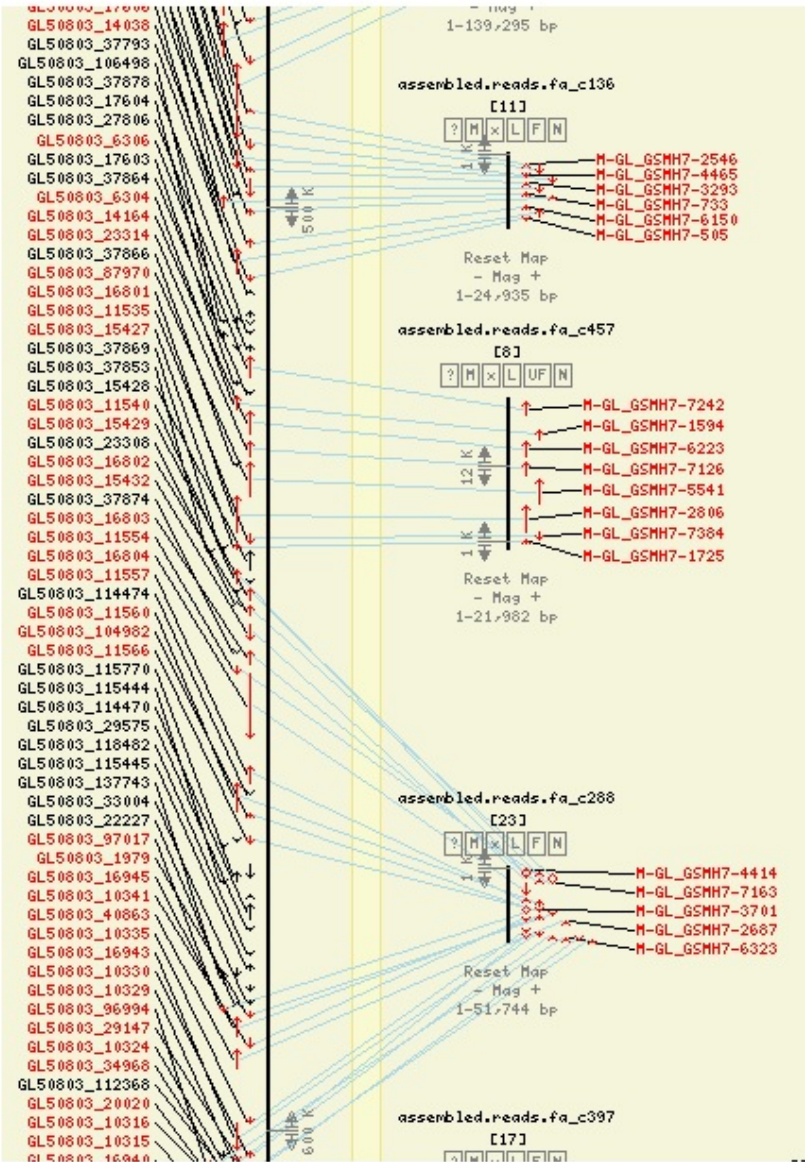

Supplement: Figure S2 — A sliding-window GC content analysis of a chromosomal region in the WB genome (CH991782) compared to the corresponding region in the GS genome. A custom script was used with a windowsize of 5000 bp and 500 bp steps. (0.20 MB PDF) [file ppat.1000560.s002.pdf]

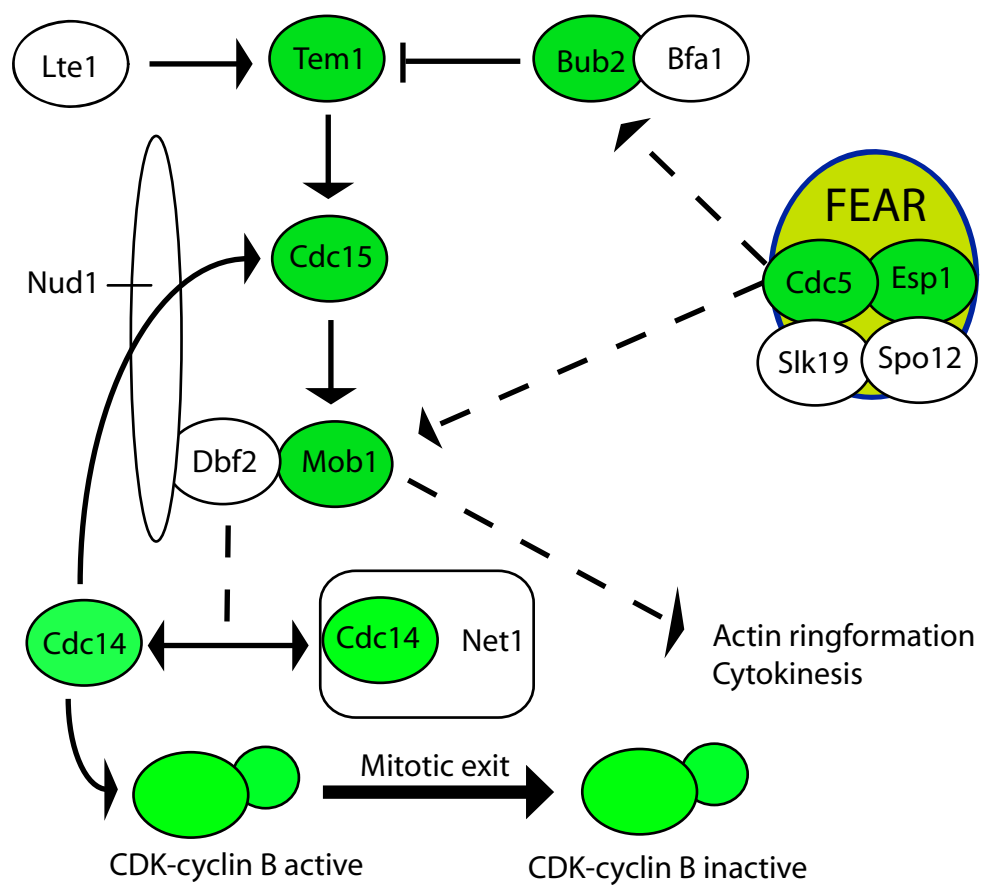

| Protein            | WB-C6 ORF      |
|--------------------|----------------|
| Tem 1-like protein | GL50803_9778   |
| Bub2               | GL50803_15248  |
| Cdc15              | GL50803_16834  |
| Mob1               | GL50803_11044  |
| Mob1               | GL50803_4008   |
| Cdc14              | GL50803_9270   |
| Cdc5               | GL50803_16835  |
| Cdc5               | GL50803_104150 |
| Esp1               | GL50803_90767  |
| Cyclin B           | GL50803_3977   |

Supplement: Figure S3 — Regulatory cytokinesis proteins in G. intestinalis and S. cerevisae. White indicates non-identified proteins in Giardia and green identified orthologs. A signaling cascade results in the assembly of an actinomyosin ring and cell division. (0.47 MB PDF) [file ppat.1000560.s003.pdf]
