# Supplementary material for: Draft Genome Sequencing of Giardia intestinalis Assemblage B Isolate GS: Is Human Giardiasis Caused by Two Different Species?
Source: PLoS Pathog. 2009 Aug 21;5(8):e1000560. doi: 10.1371/journal.ppat.1000560 (PMC2723961; doi:10.1371/journal.ppat.1000560)
Supplement: Table S1 — Distribution of contig sizes in the assembled GS genome. (0.04 MB PDF) [file ppat.1000560.s004.pdf]

Supplemental Table 1. Distribution of contig sizes in GS.

**Size (bp)**	**Number of contigs**	**Total length (kbp)**
< 100	0	0
>100 to <= 1000	2252	938.962
>1000 to <= 5000	359	702.334
> 5000 to <= 10000	65	483.362
> 10000	256	8877.131
**Total**	2932	11001.789
