# Supplementary material for: Draft Genome Sequencing of Giardia intestinalis Assemblage B Isolate GS: Is Human Giardiasis Caused by Two Different Species?
Source: PLoS Pathog. 2009 Aug 21;5(8):e1000560. doi: 10.1371/journal.ppat.1000560 (PMC2723961; doi:10.1371/journal.ppat.1000560)
Supplement: Table S3 — Small RNAs identified in Giardia GS and WB. (0.12 MB PDF) [file ppat.1000560.s006.pdf]

Small RNA name	Annotation	Type	Size (bp)	GS Identity (%)	Sequence WB	Reference
**Candidate 1**	Possible new C/D-box snoRNA	sRNA	65	94	AAAAAATAAATGAAGACAGAACCACAGACCTGTACTGACCCTTGATGTTAGTTGTCGCTCTGATA	(1)
**Candidate 2**	Possible new C/D-box snoRNA	sRNA	61	97	TGATGATTCGAATTACCGCCCGAGGGCCCTCGGGCTCCGCTGAGGACATGCTGGTCTGACT	(1)
**Candidate 3**	No annoation	Unknown	106	-	CCGATCGAAGACCAAGCGGTGCTAGGTTCAAGCCAGGGCCAAGACCCGGGCAGTCTGTGCTGTGGGGCGCCGCTGTAGACGTCTTCCGAACACACCTGCGATAAAC	(1)
**Candidate 4**	No annoation	Unknown	73	-	CCACACAAAAGGTGAGCGCGTAAGCAAAAGCCAGAAGCCCGTTGCAGCGCTTGCTTCGCAGCTCTACGGGCGC	(1)
**Candidate 5**	No annoation	Unknown	47	94	CCCCATGCATTTTCCTTGCCCAGTCTGCCTCCATACTAATTTCTCCT	(1)
**Candidate 6**	Fragment of a transcript containing RNaseP and GlsR15 snoRNA	Unknown	121	88	GATGCGCCCAGGCTGACGGTAGGACGCCTAACCCGATTCAGACTACTCCTTGGTTCCTCGCAGAATGATTATCTGTCTCCGAGCAAGCACGACTATGAGCTTACTTATGAGATCTGACTCC	
**Candidate 7**	No annotation	Unknown	41	88	AGACAGAAGTAGAGCCCGTTCTCCAGTAAATCTGCAGCTCA	(1)
**Candidate 8**	No annotation	Unknown	43	-	GCTGAGAACGTCAGGAAGGAGCCTAGAAAAGAAGTTGCTGCAC	(1)
**Candidate 9**	No annotation	Unknown	72	-	AGGAACCTATATTAGCAGAATTGGAAACGTTATAAGTGGGCTCCATCTTTTGCAGAATGTTGGAAATGTAGT	(1)
**Candidate 10**	No annotation	Unknown	86	81	ACGGGAATAACGCCCACAGGATCTCAAGGAAGGGGCCCCTCAACTTGAAGCTCTGATCGGGTCCCAAGCACAAGTAAATAATTGCC	(1)
**Candidate 11**	No annotation	Unknown	95	93	CTAGGCTGAAGCTGCCAAGGTGCGTGATCCCTCGGTGATGCCTTGAGTGTTGCTTCACCAAAGAACAACCACACGGCACAGCCGAATCTCTCATT	(1)
**Candidate 12**	No annotation	Unknown	62	95	CCCGATGACGAATAGCTGTCCTGGCGGAGGCGGTCATGACGACGAAGCCATCACGTAGGATC	(1)
**Candidate 13**	Fragment of new C/D-box snoRNA	sRNA	58	95	CACGACGGTCTACTGAGAACCCAGTATCTTTAGACTGCTGAGACAGTGTTATATGATT	(1)
**Candidate 14**	No annotation	Unknown	136	95	CAGAGTCGGCTTCGACTTTAGCGTAGTTACTGTTTCGTCGGCTTAACCGCCGATCCACTACATGCAAGGGGCAGCCGGGCTGTGAGGCAGCTGCCAGGATGGTCCTGCCCTTGTCCCGGCTGGCGCCGTCCACCTT	(1)
**Candidate 15**	No annotation	Unknown	92	97	CTTCAACTCAGCCGGACAGCCGGAGGCCGGAGACGGAGCACGGTCAGGCGGGCGGGGTGCAGTGCCAGCCCCAGCCGCAGAGCGGCTTCCTT	(1)
**Candidate 16**	Possible new H/ACA box snoRNA	Unknown	78	91	CTGCGCTCTGCCAGATACGCCGACAGAAAGCACCAAGGAAGGATGTGGATCTCCATGTCTGCCGTGTGCGCGCATATC	(1)

**Candidate 17**	No annotation	Unknown	166	98	TTCGGGATCAGTTTTGGAGTTAATACCACCAAACCCCTGTGCGTACATGTCGCCCCCTAACCTTCTGATGCGGATACCTTGCCGCAGGGCCGTTAAGCGAGGCTTGGCCCGTGCGACGATGAGGCTCCCTGCGGGGAAGCCCTGCGGCGCGTCTTAAGGAGGCAAC	(1)
**Candidate 18**	No annotation	Unknown	120	91	TCTGGATTCCGGGGAGTGTCTGGTGCCGATCGGACACTCCCTAGCCGCCACACTGACAGTTATGGTTGCAGGACAAGCTTAGCGAGTCCGAACTCGACAGGGATACTCTACAGCGTTCCT	(1)
**Candidate 19**	No annotation	Unknown	42	-	GCGTAAGGTTTTCTGTCAGACTACCCAGAGTAAACCGGTGAG	(1)
**Candidate 20**	No annotation	Unknown	133	84	TGTAGGTCTAACATGCTTGCCACGGCGTCCCCGGACATGGCACCGTCTATGTCCTGCTTGTTGGCGAGGATGAGGATGGGAACACCTGAGCTTGGGGCTGTTAGTACGCCCTCAAGAGCCGTCCGAGCCTCCT	(1)
**Candidate 21**	No annotation	Unknown	114	-	TGGACGATGAACTGGAGATGCTGGACACGGCTTTGCTCTCCCACCGGAGCACATATGCTGCAGGATGACCGGCGCCTGTCTCCCACCACGTGCCAGCTAAACTGCAGCCACATT	(1)
**Candidate 22**	No annotation	Unknown	60	-	GTATGCTGCTATGCTGACATGCCGGTACACTTTTTATGAGAGCGAATGTAAATAGCCCTG	(1)
**Candidate 23**	No annotation	Unknown	54	85	TACCACTCTGACCGTGAGGCGCATGCCTAGGGCATGGAGAAGAGCAGACTTGAG	(1)
**Candidate 24**	No annotation	Unknown	110	82	TGTGCCACTGTGGCTTCGAGCTCTATAATGCGCGACTTAAGAACCTCTGCTTCTACAGACTTTACTTCAAGTAAAGATGTCGCAGTTAGTGCCTCCTCAACACAGCTTTC	(1)
**Candidate 25**	No annotation	Unknown	140	-	GAGGTAATAGACCAGGCTGCCAGCCCGGCGAAGGTCTGCAAGTGTGACGGAGACAATGGCTACACGCTCCAGGGCGACGCGTGCACCAAGGCGGCTCCTGACAACGCGTGCCAGACCCTGGGAACCGCCGGGTGTGCCAC	(1)
**Candidate 26**	No annotation	Unknown	71	-	CTGAACGATAGAAAAGACGCGTGCGAGGCGGTTGCCAACACAAACTGCGCACAAGAGCTGCAGAATGCGGC	(1)
**Candidate 27**	Fragment of variant surface protein	Unknown	51	84	TGCCTCACCTGCACCACTTCGTCGCATAAGATCAGGCCGGACGAGAAGGGC	(1)
**Candidate 28**	No annotation	Unknown	90	-	CAGAAGAATGCCAGCAAGTCATGCAATGCCTGTGGATCCGTCCTTCGACCTTCTCCTGACAGACATGTGTCTTTTGGCATGCAGCCCTGC	(1)
**Candidate 29**	No annotation	Unknown	87	-	GCTCCTAGAGGAAGAGGCAGGCATGCAGGATATTTTTGGATGGACAGCCCTCATAAGGGCAGCAGAGAGTGGCCACGCAGGCTGCAC	(1)
**Candidate 30**	No annotation	Unknown	96	-	CCCACCGGCGTTCCAGTGCTGGCCAGGGGCAAGGAGGCCTGCTCTCCCTGGCCTCTGCGGAAACGGGCAGCTGCGTGATCCACTGACAGCCACCAC	(1)
**Candidate 31**	No annotation	Unknown	60	-	GGCGCAGACAACAGCAAGAGTCCAGATGGAGTACCTGCACTCCGCCAAGGTTTAGCGTAG	(1)
**GLsR1**	C/D-box sRNA (RNA H)	sRNA,	85 [80]	88	GTCCACTGGCCTCTCCTGAGGCAGATGATGACTTTGCGACGGGCGGACGGAGGGACGCGTGACGAAGTTTGTCGTATTCTGAATT	(2),[3]
**GLsR2**	C/D-box sRNA (RNA J)	sRNA	104 [116]	99	TGTAGCGAACCCACGCGCAAGCGTTGCTACGAGGCGATGGAGACAAAAGCAGTTACGTTCGCAACTCTCTGAGGGTTCCTGATGCTTCCTTGGATGTCCGAGCC	(2),[3]

**GLsR3**	C/D-box sRNA	sRNA (3' fragment of 5S RNA)	79	99	CCGAATTAAGAAGAGAAGCGCCGTCAGGCCCCGCCAGTACTGCGATCGGAGACGTCGTGGGAACACGGGGTGCCGAACC	(2)
**GLsR4**	C/D-box sRNA	sRNA	61	84	TGTCTCCATGACGAGAATTACGCCGCCCCAGTCTGACCCCTGACGAACGGCTTCTCTGATC	(2)
**GLsR5**	C/D-box sRNA	sRNA	89	96	AATTAAAAGCTGTGATGACAGGTTCTTGCCCCGTATGACCCTGC GATGAGTTATACAAAAGAACGCATCCAAGCCAACCGGCTGAGCTC	(2)
**GLsR6**	C/D-box sRNA	sRNA	59	93	AATGATGGCTTGTTATCCCTGTCTGAGGTCAATACCTTGATTAGACGATTTGACAGAGC	(2)
**GLsR7**	C/D-box sRNA	sRNA	60	85	CCGCGATGATTACCGAATCACAGCGATACACGATGAAGCACTCATAGTTACTCTGAGCGG	(2)
**GLsR8**	C/D-box sRNA	sRNA	70	97	CGTAGATGAAGAGAGATAAATCAGCTACCGCTGAGCCCAACGTGAGGAAGAAACCGCCTTTCGTCTGACC	(2)
**GLsR9**	C/D-box sRNA	sRNA	79	94	TAGCAACCCGTGATTTGCAACGCTTAGTCCGTGTTTCGGAGTGTCTTGCACGCTGATGAGTGAAAGCACACATGAGGTT	(2)
**GLsR10**	C/D-box sRNA	sRNA	68	91	AGAATGATGAGACGTGTTCCTCTCTCCTACAGACTCCCTGGGGATGCTATGTACACCTTACTGATTTA	(2)
**GLsR11**	C/D-box sRNA	sRNA	143	91	GGCGGTGATTAGGCTGCGTGGCGCAGCCGTGCCTTCCGCTCCGCCTGTGTCCTGGGGATTGCTCAGTGCCCCGGATCCTCACCACGAGTAAACCCGCGGCTCAAAGAAGCGCATTATCCCAGGAGGTCTAGAAACTGACCTCC	(2)
**GLsR13**	C/D-box sRNA	sRNA	100	92	ATCCATTCGTATGAGATATGATGATTGGGAGCGACCTATCTTGAGGACGACGGCCGCCCGTCTTACCTTGTGACGTTTGCCGTCTTACAATGCTCTGACC	(2)
**GLsR14**	C/D-box sRNA	sRNA	69	93	AAATGATGACAATGCGCATTTGTCAGAAGGCTCACTTCTGATGATTCCTCTGTCCATTCCCCTGATCCT	(2)
**GLsR15**	C/D-box sRNA	sRNA	87	86	GATTCAGACTACTCCTTGGTTCCTCGCAGAATGATTATCTGTCTCCGAGCAAGCACGACTATGAGCTTACTTATGAGATCTGACTCC	(2)
**GLsR16**	C/D-box sRNA	sRNA	77	92	TAAAACTATGATGAGGTTAGCGATCCCAAGCGGGGCCTGGCCCTGCGTTGCAGACAACGCATCACCGCTCTGACCTT	(2)
**GLsR17**	C/D-box sRNA (RNA D)	sRNA	144 [147]	92	GTTTCTAGACCTCCTGGGATAATGCGCTTCTTTGAGCCGCGGGTTTACTCGTGGTGAGGATCCGGGGCACTGAGCAATCCCCAGGACACAGGCGGAGCGGAAGGCACGGCTGCGCCACGCAGCCTAATCACCGCCCCTATAGTC	(2) ,[3]
**GLsR18**	H/ACA sRNA	sRNA	109	84	CGTGGCCGGTGCAGCTGCCCGCTGGCGCTTGCGAGCGTGCACAGGCCTACATCCAGGGTCATAGGTGGGGAGCGGATCCCGTCCATCCTCAATCCGGGCCCGCACAGTC	(2)
**GLsR19**	H/ACA sRNA	sRNA	176	79	AAAAACGATGATAGGGACAGGCTGGGTGGAGGCTCGGCGTCTCGTTCTGGGAAAAGCAAGCAGAAGCCCAGTTTGGTCTCTACCGGCGTATGCATGTGCATAGGCTGGCCAAGCATCGTTGATAGAAGCTGCTCTTGGTCACCGGAGGGTCTCCGGTTTCATACGCAGAGACATCC	(2)
**GLsR20**	H/ACA sRNA	sRNA	108	87	AAAAATGCCAGCTGAGTTACGTCTGTGTGCACAGGCGCGTCAGAGGCCGGCTAGAGCGCGACTGGTTGAGTTCCCAGAGCGATCTGGGTGATTAGCAGTCATACAGTC	(2)

**GLsR21**	H/ACA sRNA	sRNA	112	87	AAAACCAATATTACTACCATCGGTCCTCACCACTAGATCGGTGTTATGCTTTGTTGGGATAGCAGGC<br>CGTGCCAGTTGGACAGCCAAGGTCCACCTCTGGTTCGGCACACAT	(2)
**GLsR22**	H/ACA sRNA	sRNA	118	82	ACGCAAGCCCTCTAGCAAGATGCAGGCCGGAGCCTGTGTCTCGTTCCCTGGGGCGATAGCTCTTGCT<br>GGCAGGTCTTGCAGTGTCCATACCCGGGCAACACGTTTTCCAGCTACACCT	(4)
**GLsR23**	H/ACA sRNA	sRNA	118	81	AAAAATCGTCTGGCCGCCTCGTGCCTACGATGGGCTAGGGAAATGCCGTGACGAGACACGCACTGGG<br>TGGCCATTGCGTCTGCGGTAGATCCGCCGATTCCACAGCCCAGAAACACCC	(4)
**GLsR24**	H/ACA sRNA	sRNA	125	78	ATAATTTCGGGCAGTCCTTGCCCGCGGAGGGCACTTAAGGCTCCGGGGCCCGGGGCAGAGTCGGCCC<br>TCCCAGAGCCCGCCGACGCCCCCGAGCGCCAGCCCGGCGCAGGGGCCCGGCCACACTC	(4)
**GLsR25**	H/ACA sRNA	sRNA	119	82	TAAATCGAGGCTGCTAAAACACAGGGCTGCACAGCATCCTTGCACCTGCGTAGCCGATAGGTACGGG<br>T<br>GACCGTTTATCCCGGGCTCGTGTGGGCCCGGGTAGGCACGGTCAAAGAGTT	(4)
**Ribonuclease P RNA subunit**		ncRNA	246	89	AGGAATTAGGAGGGGCGCCACCGACCGCGGTCGGGGGAAGGTCTGAGGTCATTTTCCGTGAGGGAGG<br>C<br>GCGCCAACGCGTGCAGCAGTCGCACCAGAAACCAAGAGCCTCTGTGCTTATGCCAGGAGGAGGGCCT<br>T<br>GCCCGACTGAGAGTGCTCGCTGAAAGAGGCTGCGACGCGGGTTATTCAGTTCGATGCGCCCAGGCTG<br>A<br>CGGTAGGACGCCTAACCCGATTCAGACTACTCCTTGGTTCCT	(5)
**Signal recognition particle RNA subunit**		ncRNA	170	92	GGACGCCGAAGCAGCCGCGGCCTCTGGCTTGGACCCCGTGGCGTCGCCGGCCTCCGCGGAGGCAGGG<br>G<br>CCGGCCCGCCTTCAACTCAGCCGGACAGCCGGAGGCCGGAGACGGAGCACGGTCAGGCGGGCGGGGT<br>G<br>CAGTGCCAGCCCCAGCCGCAGAGCGGCTTCCTTT	Unpublished
**U1 RNA**	Candidate spliceosomal RNA	ncRNA	122	77	AAACATCAGCGGCATCGTCATCA(C/T)GAAGATGAGCAAAAGCATAAAGTTCGAGATC(C/T)TCA<br>T<br>CGTGTCTGCGAAGAGGAGGTTGACCAGGTTGCCGGCGGCAGAATTTTGGCGGGTGATGTCCG	(6)
**U2 RNA**	Candidate spliceosomal RNA	ncRNA	174	66	ACTTGCGTCGAACCACAGCTGCATTGAACAATAGTTTCTGCTCAAATGAGAGATCAGTATAATATGG<br>CTGATTAGCGTGCAGCTGCATGCCTTTCATATTCGTTTGTTTGTTTGCTTGTTTGTTTTAAACTAAC<br>AACTAGGATAGTCGCCTTGCAGCGACAAGAATATCCTACG	(6)
**U4 RNA**	Candidate spliceosomal RNA	ncRNA	133	92	AATATTGCGAGAAAACCCTCTTAGAATTGATAGAAGACAGTCCTGGCGGGATTCCAATAGAAACTGT<br>TAAGCTTCTAACCTTTCAGATGCTTCGTGGTGTCGAATTTTTGTGGGAGTTCATGGAGATATGTCA	(6)
**U5 RNA**	Candidate spliceosomal RNA	ncRNA	105	85	ACAACCTGCAGATCATTCATCTCTGCGGTGGATGTATCTATCCTGGTACGAGATATGTTGGGAGAGG<br>AAATGGCAGACAGTTGCATTTTTTGGGGTTATGGGCTG	(6)
**U6 RNA**	Candidate spliceosomal RNA	ncRNA	117	75	GAAGTGTCCGGGAACAAGTGAGGCCTGCACTTTTCTGCAAACAGAGGAAGTTCAAGCTGTTCGTGCA<br>TTGAGTATATTACTACAGAGTCGTGGTACTCAGACCCTACAGTGTCCTCT	(6)

References:

1. Combined experimental and computational approach to identify non-protein-coding RNAs in the deep-branching eukaryote Giardia intestinalis.
Chen XS, Rozhdestvensky TS, Collins LJ, Schmitz J, Penny D.
Nucleic Acids Res. 2007;35(14):4619-28. Epub 2007 Jun 22.
PMID: 17586815

2. Identification of 20 snoRNA-like RNAs from the primitive eukaryote, Giardia lamblia.
Yang CY, Zhou H, Luo J, Qu LH.
Biochem Biophys Res Commun. 2005 Mar 25;328(4):1224-31.
PMID: 15708007

3. Characterization of putative small nuclear RNAs from Giardia lamblia.
Niu XH, Hartshorne T, He XY, Agabian N.
Mol Biochem Parasitol. 1994 Jul;66(1):49-57.
PMID: 7527125

4. Identification and evolutionary implication of four novel box H/ACA snoRNAs from *Giardia lamblia*.
Luo J, Zhou H, Chen CJ, Li Y, Chen YQ, Qu LH.
Chin. Sci. Bull. (2006) 51:2451–2456

5. Structural implications of novel diversity in eucaryal RNase P RNA.
Marquez SM, Harris JK, Kelley ST, Brown JW, Dawson SC, Roberts EC, Pace NR.
RNA. 2005 May;11(5):739-51. Epub 2005 Apr 5.
PMID: 15811915

6. Computational identification of four spliceosomal snRNAs from the deep-branching eukaryote Giardia intestinalis.
Chen XS, White WT, Collins LJ, Penny D.
PLoS ONE. 2008 Aug 29;3(8):e3106.
PMID: 18769729
