# Supplementary material for: Draft Genome Sequencing of Giardia intestinalis Assemblage B Isolate GS: Is Human Giardiasis Caused by Two Different Species?
Source: PLoS Pathog. 2009 Aug 21;5(8):e1000560. doi: 10.1371/journal.ppat.1000560 (PMC2723961; doi:10.1371/journal.ppat.1000560)
Supplement: Table S4 — Identified synteny breaks in the GS compared to the WB genome. (0.09 MB PDF) [file ppat.1000560.s007.pdf]

GS Contig	Primers	Type of rearrangement	Genes involved	WB scaffold	Verified
**3057**	197-F1, 197-R1	Intrachromosomal translocation		CH991779	Yes,PCR and sequencing
	197-F1, 197-R2			CH991779	Yes, PCR
**387**	135-F1, 135-R1	Insertion of ORF in GS/M (GSMH7-1637) or deletion in WB/C6		CH991814	Yes, by PCR
**3089**	80-F1, 80-R1	Cluster of genes absent in GSM	GL50803_137608 VSP with INR<br>GL50803_39305 Hypothetical protein<br>GL50803_92835 VSP, putative<br>GL50803_113491 VSP<br>GL50803_41476 VSP<br>GL50803_125106 Hypothetical protein<br>GL50803_96055 VSP, putative	CH991779	Yes, PCR and sequencing
**3091**	249-F1, 249-R1	Inversion		CH991768	Yes, PCR and sequencing
**3082**	288-F1, 288-R1	Cluster of genes absent in GSM	GL50803_106010 Hypothetical protein<br>GL50803_137742 Kinase, NEK<br>GL50803_118483 Hypothetical protein<br>GL50803_114470 High cysteine membrane protein Group 6<br>GL50803_29575 Hypothetical protein<br>GL50803_118482 Kinase, NEK<br>GL50803_137743 Hypothetical protein<br>GL50803_137744 VSP	CH991782	Yes, PCR and sequencing
**111**	11-F1, 11-R1	Cluster of genes absent in GS/M	GL50803_93742 Variant-specific surface protein<br>GL50803_104193 Hypothetical protein<br>GL50803_31839 Hypothetical protein<br>GL50803_7629 Hypothetical protein<br>GL50803_93743 Hypothetical protein<br>GL50803_7632 Hypothetical protein	CH991801	Yes, PCR and sequencing
**1168**	211-F1, 211-R1	Partial deletion of gene	GL50803_16318 High cysteine membrane protein Group 1	CH991767	Yes, PCR and sequencing
**419**	138-F1, 138-R1	Intrachromosomal translocation		CH991767	Yes, PCR and sequencing

**112**	110-F1, 110-R1	Cluster of genes absent in GS/M	GL50803_32958 Protein kinase<br>GL50803_4033 Protein kinase<br>GL50803_92983 Protein 21.1<br>GL50803_13520 VSP<br>GL50803_92985 Hypothetical protein<br>GL50803_20912 Hypothetical protein	CH991779	Yes, PCR and sequencing
**3092**	556-F1, 556-R1	Inversion and cluster of genes absent in GS/M<br>(other edge in contig244)	GL50803_103540 Hypothetical protein<br>GL50803_27813 Protein 21.1<br>GL50803_Hypothetical protein<br>GL50803_Hypothetical protein	CH991763	Yes, PCR and sequencing
**3092**	244-F1, 244-R1	Inversion and cluster of genes absent in GS/M (other edge in contig556)		CH991763	Yes, PCR and sequencing
**647**	159-F1, 159-R1	Two genes absent in GS/M	GL50803_31542 Hypothetical protein<br>GL50803_14324 High cysteine protein	CH991782	Yes, PCR and sequencing
**1538**	245-F1, 245-R1	Interchromosomal translocation		CH991768<br>CH991767	Yes, PCR and sequencing
**3093**	118-F1, 118-R1	Gene absent in WB/C6	M-GL_GSMH7-2323 Hypothetical protein<br>(Unique protein distantly related to Kinase, NEK)	CH991814	Yes, PCR and sequencing
**3081**	113-F1, 113-R1	Cluster of genes missing in GS/M	GL50803_1713 Protein 21.1<br>GL50803_24701 Hypothetical protein<br>GL50803_115673 Hypothetical protein<br>GL50803_117312 Hypothetical protein<br>GL50803_11521 VSP<br>GL50803_115439 Hypothetical protein<br>GL50803_106057 Hypothetical protein<br>GL50803_118786 VSP, putative<br>GL50803_115438 Hypothetical protein<br>GL50803_117311 Hypothetical protein<br>GL50803_115672 Hypothetical protein	CH991768	Yes, PCR and sequencing
**2481**	467-F1, 467-R1	Cluster of genes missing in GS/M	GL50803_102841 Hypothetical protein<br>GL50803_31911 Hypothetical protein<br>GL50803_42698 Kinase, NEK<br>GL50803_95847 Protein 21.1<br>GL50803_113433 Hypothetical protein<br>GL50803_8595 VSP	CH991763	Yes, PCR and sequencing

**3067**	C202F, C202R1	Interchromosomal translocation		CH991768<br>CH991767	Yes, PCR and sequencing
**1431**	235-F1, 235-R1	Interchromosomal translocation		CH991762<br>CH991771	Yes, PCR and sequencing
**3095**	326-F1, 326-R1	Cluster of genes missing in GS/M	GL50803_102444 Protein 21.1<br>GL50803_35621 Hypothetical protein<br>GL50803_112518 Kinase, NEK<br>GL50803_112519 Hypothetical protein	CH991767	Yes, PCR and sequencing
**3065**	237-F1, 237-R1	Insertion of three ORFs in GS/M (GSMH7-2671, -6128 and 1584 (Cathepsin B) or deletion in WB/C6		CH991782	Yes, PCR product (mixed)
**3091**	249-F3, 249-R3	Inversion		CH991768	Yes, PCR product (mixed)
**3050**	83-F1, 83-R1	Insertion of VSP in GS/M (GSMH7-4823) or deletion in WB/C6		CH991763	Yes, PCR product (mixed)
**1987**	286-F1, 286-R1	Cluster of genes missing in GS/M	GL50803_112693 VSP<br>GL50803_112695 Hypothetical protein<br>GL50803_112696 Hypothetical protein<br>GL50803_98653 Hypothetical protein	CH991768	Yes, PCR product (mixed)

Putative misassembled regions

**3046, 3047**<br><br>**3044, 3045**	474-F1, 474-R1<br><br>455-F1, 455-R1	Synteny break in highly similar genes Protein disulfide isomerase	Miassembly between PDI-3, GL50803_14670 and PDI-5, GL50803_8064		Verified and corrected
**1036**<br><br>**2609**	PeroxiF,C196R<br><br>PeroxiF, C592F	Synteny break in highly similar genes Peroxiredoxin 1	Putative missassembly between GL50803_14521 and GL50803_16076	CH991782<br><br>CH991776	Not verified
**1055**	2-F1, 2-R1	Synteny break in highly similar genes Hypothetical protein	Putative missassembly between GL50803_115478 and GL50803_115479	CH991793	Not verified
**2731**	71-F1, 71-R1	Synteny break in highly similar genes Topoisomerase II - like	Putative missassembly between GL50803_16795 Topoisomerase II and GL50803_101906 SDK1		Not verified

**2460**	445-F1, 445-R1	Synteny break in similar genes<br>ABC transporter family proteins	Putative missassembly between GL50803_92223 and GL50803_8227		Not verified
**2493**		Synteny break in highly similar genes<br>Acyl-CoA synthetase	Putative missassembly between GL50803_16667 and GL50803_86511		Not verified
**3060, 3076**	191-F1, 191-R1	Synteny break in highly similar genes, Histone H2A			Not verified

Regions that have not been verified

**3079**		Insertion of ORF in GS/M (GSMH7-5233) Na+ driven multidrug efflux pump or deletion in WB/C6		CH991762	Not verified
**2358**		Insertion of ORF in GS/M (GSMH7-2895) Protein 21.1 or deletion in WB/C6		CH991782	Not verified
**757**	169-F1, 169-R1	Inversion		CH991779	Not verified
**3091**	249-F2, 249-R2	Inversion		CH991768	Not verified
**1055**	2-F2, 2-R2	Synteny break			Not verified
**485**	144-F1, 144-R1	Intrachromosomal translocation		CH991768	Not verified
**2435**	42-F1, 42-R1	Problematic region		CH991782	Not verified
**2435**	42-F2, 42-R2	Problematic region		CH991782	Not verified
**3048, 3049**	621-F1, 621-R1	Synteny break			Not verified
**3054**	69-F1, 69-R1	Deletion of GL50803_119672 in GS/M			Not verified
**1420, 3063**	F10217-F, 234Link1866	Should be paired			Not verified
