# Supplementary material for: Draft Genome Sequencing of Giardia intestinalis Assemblage B Isolate GS: Is Human Giardiasis Caused by Two Different Species?
Source: PLoS Pathog. 2009 Aug 21;5(8):e1000560. doi: 10.1371/journal.ppat.1000560 (PMC2723961; doi:10.1371/journal.ppat.1000560)
Supplement: Table S5 — GS supercontigs generated using PCR and Sanger sequencing. (0.04 MB PDF) [file ppat.1000560.s008.pdf]

Supercontigs

Supercontig name	Size (bp)	Mended genes	Contigs joined	New contig
Supercontig 1_1	40600	GL50803_15295 Zinc finger protein	c429<br>c584<br>c395	3079
Supercontig 1_2	23426	GL50803_10829 Glucosamine-6-phosphate deaminase	c204<br>c222	3090
Supercontig 1_3	29660	GL50803_15156 SRP GTPase	c755<br>c207	3080
Supercontig 2	136041	GL50803_16733 Kinase, NEK-frag<br>GL50803_13475 Axoneme-associated protein GASP-180<br>GL50803_89887 Nucleolar GTP-binding protein 2<br>GL50803_15344 MCM2	c492<br>c427<br>c97<br>c484<br>c249	3091
Supercontig 3	131258	GL50803_5710 Hypothetical protein<br>GL50803_15247 Grp94/Hsp90	c244<br>c405<br>c556<br>c504	3092
Supercontig 4	139328	GL50803_114210 Hypothetical protein<br>VSP??	c498<br>c633<br>c113<br>c19	3081
Supercontig Jon	65942	GL50803_137730 Kinase	c276<br>c468	3086
Supercontig Per 2	101072	GL50803_11690 Variant-specific surface protein	c176<br>c154	3088
Supercontig 10	65800	GL50803_13200 RNA helicase, putative	c77<br>c326	3095
Supercontig Per 1	128690	GL50803_16653 Hypothetical protein	c58<br>c312<br>c370<br>c616<br>c100	3087
Supercontig 6	99476	GL50803_135231 Histone H3	c118<br>c578	3093
Supercontig 7_1	51329	GL50803_27735 Mre11	c461<br>c193	3094
Supercontig 7_2	73812	GL50803_15428 IFT complex B	c288<br>c457	3082
Supercontig 7_3	57005	GL50803_17188 Kinase, NEK	c318<br>c594	3083
Supercontig 7_4	103750	GL50803_8610 Coiled-coil protein	c239<br>c296	3084
Supercontig 8_1	10813	GL50803_16110 tRNA-ribosyltransferase, putative	c674<br>c277	3085
Supercontig 8_2	105695	GL50803_9922 Exonuclease 1	c258<br>c80	3089
17 Supercontigs	**1363697**	At least 21 genes mended	44 contigs	
