# Supplementary material for: Draft Genome Sequencing of Giardia intestinalis Assemblage B Isolate GS: Is Human Giardiasis Caused by Two Different Species?
Source: PLoS Pathog. 2009 Aug 21;5(8):e1000560. doi: 10.1371/journal.ppat.1000560 (PMC2723961; doi:10.1371/journal.ppat.1000560)
Supplement: Table S7 — Supplementary Giardia GS MIGS report. (0.07 MB PDF) [file ppat.1000560.s010.pdf]

Giardia lamblia isolate GS genome sequence report as recommended by the Genome Standards Consortium (Field, et al., Nat. Biotech. 2008).

Eukaryote:
- **ncbiOrganismName** : *Giardia lamblia* (syn. *Giardia intestinalis*, *Giardia duodenalis*) isolate GS
- **ncbiTaxID** : 5741
- **gcatID** : 005215_GCAT
- **goldStamp** : Gi00397
- **genomeProjectID** : 33815
- **studyData**:
  - **projectName** : *Giardia lamblia* isolate GS draft genome sequencing
- **originalSample** :
  - **organismalMaterial :**
    - **name** :
      - **genus :** Giardia
        - **species** : *Giardia lamblia*
      - **strain**: GS M-H7
        - **typeStrain** : false
    - **healthDiseaseStatus** :
- **Isolate :**
  - **cultureCollection** :
    - **identifier** : ATCC50581
  - **subspecificGeneticLineage** : Genotype B, M-H7
  - **trophicLevel** : heterotrophic (consumer)
  - **numReplicons** : 10
  - **bioticRelationship :** pathogen
  - **oxygenRelation** : anaerobe
  - **ploidyLevel :** diploid and binucleic
  - **extrachromosomalElements :** no
  - **estimatedSize** : 12000000
  - **originalHost** : human
  - **alternateHost** : mice and other mammals
  - **healthDiseaseStatus** : disease
  - **pathogenicity** : pathogenic
  - **propagation** : infection of the GI-tract and formation and excretion of cysts
- **nucExtract:**
  - **method** : Easy DNA kit (Invitrogen)
- **sequencing:**
  - **sequencingMethod** : pyrosequencing (454)
  - **assembly** :
    - **assemblyMethod** : MIRA sequence assembler
    - **estimatedErrorRate** : 0.29% of raw bases (before assembly)
      - **methodCalculation** : phred quality scores
  - **status** : draft
    - **coverage** : 16x
    - **contigs** : 2931
    - **accession** : ACGJ00000000
