# Supplementary material for: Draft Genome Sequencing of Giardia intestinalis Assemblage B Isolate GS: Is Human Giardiasis Caused by Two Different Species?
Source: PLoS Pathog. 2009 Aug 21;5(8):e1000560. doi: 10.1371/journal.ppat.1000560 (PMC2723961; doi:10.1371/journal.ppat.1000560)
Supplement: Text S1 — Summary of tRNA genes identified in the GS genome. (0.06 MB PDF) [file ppat.1000560.s011.pdf]

<u>*Identification of tRNA genes*</u>

1.1 tRNA-analysis-WB genome (from tRNAScan)

```
tRNAs decoding Standard 20 AA:              61
Selenocysteine tRNAs (TCA):                 0
Possible suppressor tRNAs (CTA,TTA):        0
tRNAs with undetermined/unknown isotypes:   0
Predicted pseudogenes:                      2
                                            -------
Total tRNAs:                                63

tRNAs with introns:             5

| Gln-TTG: 4 | Tyr-GTA: 1 |

Isotype / Anticodon Counts:

Ala   : 4         AGC: 2       GGC:         CGC: 1       TGC: 1
Gly   : 4         ACC:         GCC: 2       CCC: 1       TCC: 1
Pro   : 3         AGG: 1       GGG:         CGG: 1       TGG: 1
Thr   : 3         AGT: 1       GGT:         CGT: 1       TGT: 1
Val   : 4         AAC:         GAC: 2       CAC: 1       TAC: 1
Ser   : 4         AGA: 1       GGA:         CGA: 1       TGA: 1       ACT:
GCT: 1
Arg   : 6         ACG: 2       GCG:         CCG: 1       TCG: 1       CCT: 1
TCT: 1
Leu   : 6         AAG: 2       GAG:         CAG: 1       TAG: 1       CAA: 1
TAA: 1
Phe   : 1         AAA:         GAA: 1
Asn   : 2         ATT:         GTT: 2
Lys   : 3                                   CTT: 2       TTT: 1
Asp   : 3         ATC:         GTC: 3
Glu   : 3                                   CTC: 2       TTC: 1
His   : 1         ATG:         GTG: 1
Gln   : 4                                   CTG: 1       TTG: 3
Ile   : 4         AAT:         GAT: 3                    TAT: 1
Met   : 3                                   CAT: 3
Tyr   : 1         ATA:         GTA: 1
Supres: 0                                   CTA:         TTA:
Cys   : 1         ACA:         GCA: 1
Trp   : 1                                   CCA: 1
SelCys: 0                                                TCA:
```

1.2 tRNA-analysis-GS genome (from tRNAScan)

Subject: GS assembled contigs

tRNAs decoding Standard 20 AA:	58
Selenocysteine tRNAs (TCA):	0
Possible suppressor tRNAs (CTA,TTA):	0
tRNAs with undetermined/unknown isotypes:	0
Predicted pseudogenes:	0

Total tRNAs:	58

tRNAs with introns: 7

| Gln-TTG: 6 | Tyr-GTA: 1 |

Isotype / Anticodon Counts:

Ala	: 3	AGC: 1	GGC:	CGC: 1	TGC: 1		
Gly	: 2	ACC:	GCC: 1	CCC: 1	TCC:		
Pro	: 3	AGG: 1	GGG:	CGG: 1	TGG: 1		
Thr	: 4	AGT: 2	GGT:	CGT: 1	TGT: 1		
Val	: 2	AAC:	GAC:	CAC: 1	TAC: 1		
Ser	: 4	AGA: 1	GGA:	CGA: 1	TGA: 1	ACT:	GCT: 1
Arg	: 6	ACG: 2	GCG:	CCG: 1	TCG: 1	CCT: 1	TCT: 1
Leu	: 6	AAG: 2	GAG:	CAG: 1	TAG: 1	CAA: 1	TAA: 1
Phe	: 1	AAA:	GAA: 1				
Asn	: 2	ATT:	GTT: 2				
Lys	: 2			CTT: 1	TTT: 1		
Asp	: 2	ATC:	GTC: 2				
Glu	: 2			CTC: 1	TTC: 1		
His	: 1	ATG:	GTG: 1				
Gln	: 8			CTG: 1	TTG: 7		
Ile	: 4	AAT:	GAT: 3		TAT: 1		
Met	: 3			CAT: 3			
Tyr	: 1	ATA:	GTA: 1				
Supres	: 0			CTA:	TTA:		
Cys	: 1	ACA:	GCA: 1				
Trp	: 1			CCA: 1			
SelCys	: 0				TCA:		

1.3 tRNA-analysis on GS reads not in assembly (from tRNAscan)

Subject: Analysis of reads not included in the GS assembly

tRNAs decoding Standard 20 AA: 10

Selenocysteine tRNAs (TCA): 0

Possible suppressor tRNAs (CTA,TTA): 0

tRNAs with undetermined/unknown isotypes: 1

Predicted pseudogenes: 0

Total tRNAs: 11

tRNAs with introns: 1

| Gln-TTG: 1 |

Isotype / Anticodon Counts:

Ala	: 1	AGC:	GGC:	CGC: 1	TGC:		
Gly	: 3	ACC:	GCC:	CCC: 1	TCC: 2		
Pro	: 0	AGG:	GGG:	CGG:	TGG:		
Thr	: 0	AGT:	GGT:	CGT:	TGT:		
Val	: 2	AAC:	GAC: 2	CAC:	TAC:		
Ser	: 0	AGA:	GGA:	CGA:	TGA:	ACT:	GCT:
Arg	: 0	ACG:	GCG:	CCG:	TCG:	CCT:	TCT:
Leu	: 0	AAG:	GAG:	CAG:	TAG:	CAA:	TAA:
Phe	: 0	AAA:	GAA:				
Asn	: 0	ATT:	GTT:				
Lys	: 0			CTT:	TTT:		
Asp	: 1	ATC:	GTC: 1				
Glu	: 0			CTC:	TTC:		
His	: 0	ATG:	GTG:				
Gln	: 1			CTG:	TTG: 1		
Ile	: 1	AAT:	GAT: 1		TAT:		
Met	: 1			CAT: 1			
Tyr	: 0	ATA:	GTA:				
Supres	: 0			CTA:	TTA:		
Cys	: 0	ACA:	GCA:				
Trp	: 0			CCA:			
SelCys	: 0				TCA:		
